# Mutant Kras and mTOR crosstalk drives hepatocellular carcinoma development via PEG3/STAT3/BEX2 signaling

**DOI:** 10.7150/thno.76873

**Published:** 2022-11-16

**Authors:** Yuan-Deng Luo, Xiao-Yu Liu, Lei Fang, Hong-Qiang Yu, Yu-Jun Zhang, Min Chen, Lei-Da Zhang, Chuan-Ming Xie

**Affiliations:** 1 Key Laboratory of Hepatobiliary and Pancreatic Surgery, Institute of Hepatobiliary Surgery, Southwest Hospital, Third Military Medical University (Army Medical University), Chongqing, 400038, China.; 2 School of Medicine, Southern University of Science and Technology, Shenzhen, 518055, China.

**Keywords:** Kras/Mek/Erk, PEG3, hepatocellular carcinoma, STAT3, Cancer therapy

## Abstract

**Background & Aims:** Abnormal activation of mTOR through loss of tuberous sclerosis complex (Tsc) frequently occurs in hepatocellular carcinoma (HCC). Mutant Kras could induce aggressive HCCs. Here, we aim to identify the predictive or prognostic biomarkers for HCC patients with Kras mutant and mTOR hyperactivation, and to provide potential therapeutic approaches for this subtype of HCCs.

**Methods:** We generated transgenic mice in which hepatocytic mTOR was hyperactivated through Tsc1 insufficiency with or without oncogenic Kras^G12D^. Bioinformatics and gain- or loss-of-function studies were used to illustrate the mechanisms underlying oncogenic pathway alterations. Transcriptional profiling was used to identify biomarker for the subtype of HCC. The therapeutic efficacy of targeting mTOR was tested in a liver orthotropic homogeneous murine model.

**Results:** Oncogenic Kras^G12D^ facilitated mTOR activation via the Mek/Erk/ROS axis, leading to HCC tumorigenesis and metastasis. Inhibition of Mek/Erk enhanced the anticancer effect of mTOR inhibitor via reduction of mTOR activity. Paternally expressed 3 (PEG3) was responsible for Kras/Erk- and mTOR-driven HCC. Elevated PEG3 protein interacted with STAT3 and promoted its transcriptional activity, resulting in the upregulation of proliferation- and metastasis-related proteins. Targeting mTOR significantly inhibited these actions *in vitro* and *in vivo*. Moreover, in clinical samples, PEG3 was identified as a new poor prognostic marker for HCC patients with Kras/Erk and mTOR hyperactivation.

**Conclusion:** These findings reveal the underlying mechanism of hepatocytic Kras/Erk-driven mTOR activation and its downstream targets (PEG3 and STAT3) in HCC, identify PEG3 as a new prognostic biomarker for HCC with Kras/Erk and mTOR hyperactivation, and provide a potential therapeutic strategy for this subset of HCC patients.

## Introduction

Hepatocellular carcinoma (HCC) accounts for most primary liver cancers and remains a health challenge with growing incidence worldwide 1, 2. The molecular pathogenesis of HCC is extremely complex and heterogeneous 2. Although the understanding of the pathophysiology and diversity of HCC has improved, relevant data have yet to be translated into clinical practice to date. In up to 50% of HCCs, the mammalian target of rapamycin (mTOR) signaling are aberrantly hyperactivated to drive tumor progression by underwriting biosynthetic programs and promoting proliferation 3-5, which is associated with a poor prognosis, poor differentiation and earlier recurrence 5. However, HCC patients received mTOR inhibitors didn't seem to achieve desired effect 6, 7. In mammals, mTOR is divided into two distinct complexes named mTOR complex 1 (mTORC1) and mTOR complex 2 (mTORC2) 8, 9. Functionally, mTORC1 integrates nutritional and environmental information to tune the metabolic balance, while mTORC2 governs cytoskeletal behavior and activates prosurvival signaling 3, 9. mTOR is frequently activated by upstream nodes 10, 11. Among all mutant mTOR-related genes identified, tuberous sclerosis complex 1 (Tsc1) and Tsc2 have been found to be the most frequently mutated (16.2%) in HCCs 12. Loss or impaired function of either Tsc1 or Tsc2, which together act to negatively regulate mTOR signaling, leads to strong and sustained mTOR signaling activation 13.

Notably, the Ras/Mek/Erk pathway is also highly activated in 50-100% of HCCs and correlated with a poor prognosis 14. Ras is a pivotal and hypermutated oncogene in multiple cancers, including HCC 15. The activated Ras cascade launched by mutant Ras phosphorylates its downstream targets Raf/Mek/Erk and/or PI3K/Akt/mTOR to regulate cell proliferation, cycling, and differentiation, which are key oncogenic signal transduction pathways activated in HCC 16, 17. Kras is the most frequently mutated isoform of Ras, followed by Nras, and Hras 18. Thus, Kras mutants are attractive potential therapeutic targets. However, attempts to develop Kras analog inhibitors have been challenged 19. Fortunately, sorafenib was developed to block multiple kinases, including Kras downstreams Braf and Raf1 20. However, clinical practice demonstrated sorafenib is only benefit a fraction of HCC patients 21. So far, although hyperactivation in mTOR and mutation in oncogene Kras had been respectively discussed a lot in multiple malignancies, it is still unclear the synergistic effect of them in HCC, and potential therapeutic target for HCC with this molecular background.

Paternally expressed gene 3 (PEG3), a paternally expressed imprinted gene, encodes C2H2 zinc-finger DNA-binding protein 22. PEG3 can regulate the physiological processes related to energy homeostasis found in multiple tissues, such as the brain, testis, ovary, and placenta 23. PEG3 has been clarified to be a primary putative gene involved in cancer inhibitory activity in non-small-cell-lung cancer 24. Epigenetic silencing and loss of imprinting of PEG3 are implicated in glioma tumorigenesis 25. In breast cancer, PEG3 mutation was associated with a high tumor mutation burden and an inferior prognosis 23. However, the function of PEG3 in HCC has not been investigated.

Here, we found that Kras^G12D^ mutation, when cooperating with hyperactive mTOR driven by loss of Tsc1, markedly increases HCC formation and promote lung metastasis. Mechanistic studies in primary liver cells and an *in vivo* transgenic murine model indicated that Kras^G12D^ mutation further hyperactivates the mTOR pathway by Mek/Erk and elevated cellular ROS levels in response to Tsc1 insufficiency. Furthermore, primary liver cells and *in vivo* murine models demonstrated that HCC with Tsc1 insufficiency and Kras^G12D^ mutation are sensitive to mTOR inhibitors. This effect could be further enhanced by dual inhibition of Mek and mTOR. These observations have potential therapeutic implications for treating a subtype of HCCs with Kras/Erk and mTOR hyperactivation. Moreover, paternally expressed gene 3 (PEG3) is identified as a downstream target of mTOR and serves as a novel prognostic biomarker for this subtype of HCCs. Elevated PEG3 can directly bind to STAT3 and increase its transcriptional activity, leading proliferation and metastasis related genes expression, leading to tumorigenesis.

## Patients and Methods

### Patients and samples

A total of 166 human HCC specimens were obtained during surgery prior to any therapeutic intervention at the Southwest Hospital in China with approval by the Clinical Research Ethics Committee. All subjects provided informed consent for obtaining the study specimens. All specimens were formalin fixed and paraffin embedded and then saved for experiments. Data for another HCC cases were obtained from the cancer genomic atlas (TCGA), Gene Expression Omnibus (GEO) and Proteomics Identifications (PRIDE). All specimens obtained from our hospital or databases met the following criteria: (a) primary HCC only; other types, such as cholangiocellular carcinoma or mixed liver cancer, were excluded. (b) Primary HCC patients who died from other causes were excluded. (c) Primary HCC with other primary cancers was excluded. (d) Patients aged under 18 years and over 75 years were excluded. (e) Patients who were unwilling to attend appointments for this research were excluded.

### Mouse lines, breeding and experiments

Only male mice were used in this study. The liver-specific Cre recombinase mouse line *Alb-Cre* (016833) and mice containing *Loxp-STOP-Loxp-Kras^G12D^* (*LSL-Kras^G12D^*; 008179) or a floxed Tsc1 allele (*Tsc1^fl/fl^*, 005680) were obtained from the Jackson Laboratory (Bar Harbor, Maine, USA). All mice were on the C57BL/6 genetic background. *Tsc1^fl/fl^* and *Kras^G12D^* mice were bred separately with *Alb-Cre* mice to generate *Tsc1^fl/fl^;Alb-Cre* and *Kras^G12D^;Alb-Cre* mice, respectively. These two types of mice were then crossed to generate *Kras^G12D^;Tsc1^fl/fl^;Alb-Cre* mice (**Figure S1A**). All genotypes were validated by PCR using genomic DNA extracted from mouse tails and liver tissues (**Figure S1B-D**). Wild-type (WT) C57BL/6 mice were purchased from the Chinese Academy of Medical Sciences (Beijing, China). Mice were bred at the specific pathogen-free (SPF) mouse facility at the Animal Center of Army Medical University. Mice were monitored for up to 280 days. The tumor number, largest tumor size and the liver: body weight ratio were evaluated. All experiments were approved by the Institutional Animal Care and Use Committees of Army Medical University.

### Statistical analysis

R 4.1.2 software, SPSS 20.0 software (Statistical Package for the Social Sciences, IBM, New York, USA) and GraphPad Prism 8.0 software (GraphPad, San Diego, California, USA) were used for statistical analysis and data visualization. For comparing the distribution of categorical factors between different groups, the Pearson Chi-square test or Fisher exact test were used. An unpaired Student's t-test was performed to compare differences between two continuous variables. A paired Student's t-test was performed to compare differences between paired variables. One-way ANOVA was used to compare differences between different independent continuous variables with only one variable. Two-way ANOVA was used to compare differences between different independent continuous variables with two variables. Survival curves were drawn using the Kaplan-Meier method and compared by the Log-rank test or Breslow test. Univariate and multivariate analyses were conducted with Cox regression analysis. Hazard ratios (HRs) and 95% confidence intervals (95% C.I.s) were evaluated. All experiments were repeated at least three times. The sample size for animal experiments was determined using power calculations. Many statistically significant effects were observed in the data, suggesting that the effective sample size was sufficient for studies. A p value ≤ 0.05 was considered statistically significant (*p ≤ 0.05; **p ≤ 0.01; and *** p ≤ 0.001). The results are expressed as the mean ± SEM unless indicated otherwise.

### Further materials and methods

The other detailed materials and methods are provided in the supplementary materials of this manuscript.

## Results

### Mutant Kras promotes mTOR signaling-driven HCC tumorigenesis and lung metastasis

**Figure 1:**
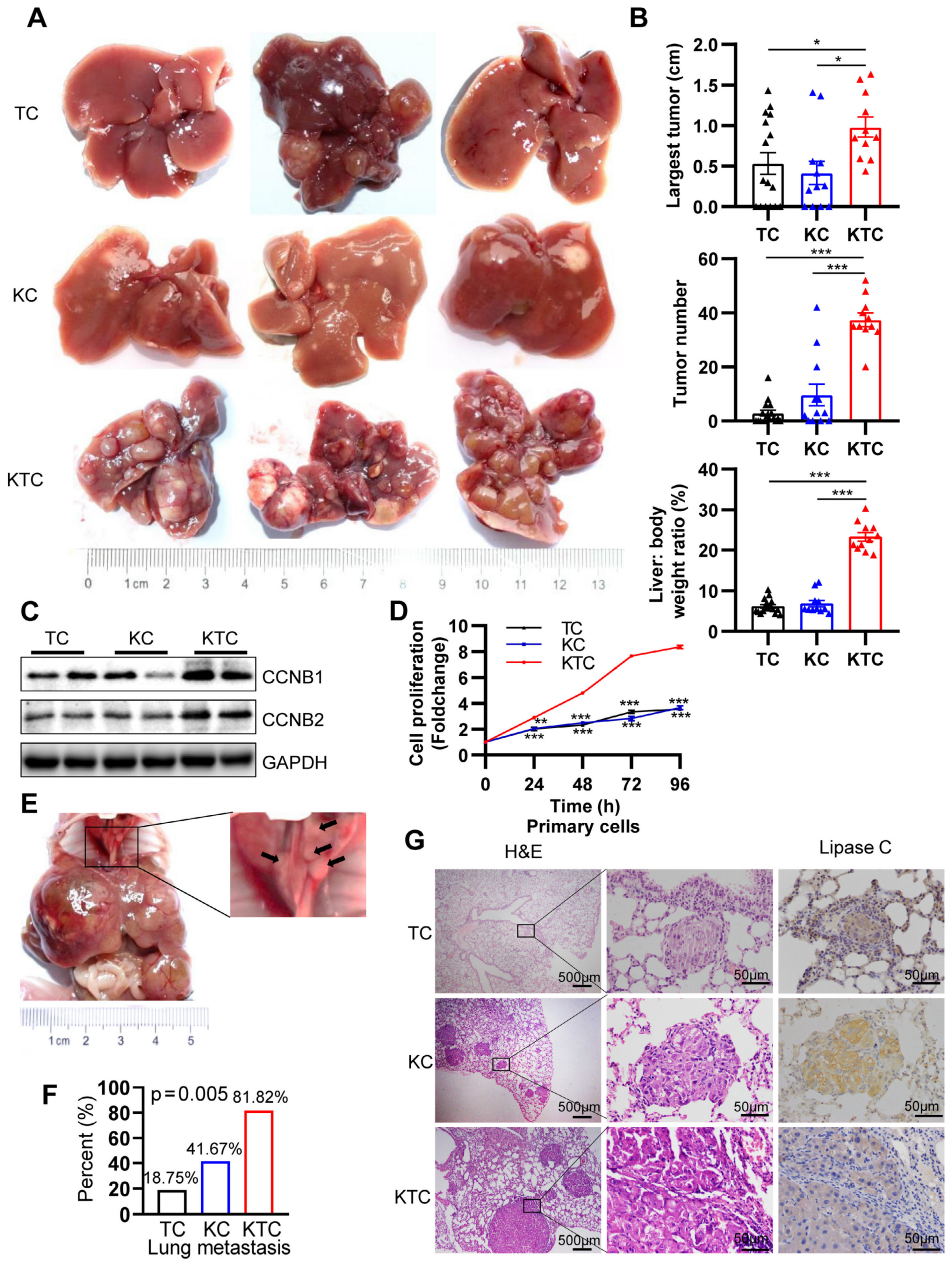
** Mutant Kras promotes Tsc1 insufficiency-driven HCC tumorigenesis and lung metastasis.** (**A**) Representative HCC tumorigenesis images of *Tsc1^fl/fl^;Alb-Cre* mice (TC), *Kras^G12D^;Alb-cre* mice (KC) and *Kras^G12D^;Tsc1^fl/fl^*; *Alb-Cre* mice (KTC) at 280 days old. (**B**) Quantification of the largest tumor size, tumor number, and liver: body weight ratio in TC (n = 16), KC (n = 12) and KTC mice (n = 11). (**C**) WB showing increased expression of CCNB1 and CCNB2 proteins in KTC mice compared with TC and KC mice. (**D**) Representative proliferation curves for primary liver cells isolated from the livers of 280-day-old TC, KC and KTC mice. (**E**) Macroscopic image of lung metastases was observed in KTC mice. (**F**) The lung metastasis rate was significantly increased in KTC (9/11) compared with TC (3/16) or KC mice (5/12) (Pearson Chi-square test, p = 0.005). (**G**) Representative H&E and IHC staining images for Lipase C showing distinct lung metastatic foci expressing Lipase C. Images were obtained at 4X or 40X magnification; scale bar, 500 or 50 µm. Data are represented by the mean ± SEM. *p < 0.05; **p < 0.01; ***p < 0.001. One-way ANOVA was used in **B**; two-way ANOVA was used in **D**.

The function of mutated Kras in mTOR-driven hepatocarcinogenesis is still unknown. G12D is the most frequently observed mutation that leads to constitutive activation of Kras in cancer cells 26. We utilized the liver-specific Kras G12D activating mutation (herein referred to as KC or *Kras^G12D^;Alb-Cre* mice) to establish constitutively active Kras^G12D^ mutation in C57BL/6 mice. Loss of either Tsc1 or Tsc2 decreases the inhibition of mTOR, leading to its activation 27. Hence, liver-specific Tsc1-knockout mice (herein referred to as TC or *Tsc1^fl/fl^;Alb-Cre* mice) were also generated. To generate mice with both activation of Kras and loss of Tsc1, we crossed mice bearing *Kras^G12D^* and *Tsc1^fl/fl^* mice (herein referred to as KTC mice, **Figure S1A**). Western blotting (WB) was used to verify that Tsc1 was knocked out at the protein level (**Figure S1E**). All animals were followed up for up to 280 days and sacrificed for histological analysis (**Figure S1A**). Compared with TC and KC mice, KTC mice developed many tumors, had larger tumors, and had a high liver: body weight ratio (**Figure 1A-B**). Regarding the tumor incidence rate, KTC mice had a 100% tumor incidence rate (11/11), whereas TC and KC mice had moderate tumor incidence rates of 62.5% (10/16, p = 0.021) and 66.67% (8/12, p = 0.035), respectively (Pearson Chi-square test, **Table S1**). Developed tumors were further validated by the expression of GPC3 (an HCC biomarker) and CK19 (an intrahepatic cholangiocarcinoma biomarker) (**Figure S1F**) 28, 29. Consistently, the levels of the proliferation markers Ccnb1, Ccnb2, Ki-67 and Pcna were significantly increased at both the mRNA and protein levels in KTC tumors compared to TC or KC tumors (**Figure 1C, Figure S2A**). And then, primary cells were isolated from the livers of TC, KC and KTC mice. Proliferation evaluation demonstrated that the KTC primary cells grew faster than the TC or KC primary cells, which was similar to the observations in murine models (**Figure 1D**).

As single Kras^G12D^ or hyperactive mTOR can often drive early metastasis leading to high mortality in malignancies 10, 30, KTC tumors were identified as more aggressive compared with TC or KC tumors alone. The aggressive tumors were characterized a marked increase in lung metastasis in 81.82% (9/11) of KTC mice, compared with 18.75% (3/16) in TC and 41.67% (5/12) in KC mice (Pearson Chi-square test, p = 0.005, **Figure 1E-F**). In addition, IHC staining of lung tumor mets for Lipase C following H&E was performed 14, demonstrating the lung tumor mets were from liver (**Figure 1G**). This finding was furtherly supported by high expression of the metastasis-related genes, *Icam1*, *Vcam1, Mmp9* and *Vim* 31, 32 in KTC compared to TC or KC mice (**Figure S2B**).

Furthermore, the fibrogenic-related genes *Col1a1*, *Col1a2*, *Timp1*, *Pdgfrb* and *Pdgfb* 33 expression were significantly increased in KTC tissues compared to TC or KC tissues (**Figure S2C**). These results were furtherly supported by Sirius red staining, which indicated high fibrosis levels in KTC mice (**Figure S2D**). Taken together, these results indicated that liver-specific Kras activation and homozygous Tsc1 deletion caused the rapid progress and metastasis of HCC.

### Kras facilitates mTOR signaling via the Ras/Mek/Erk axis but not Akt pathway

**Figure 2:**
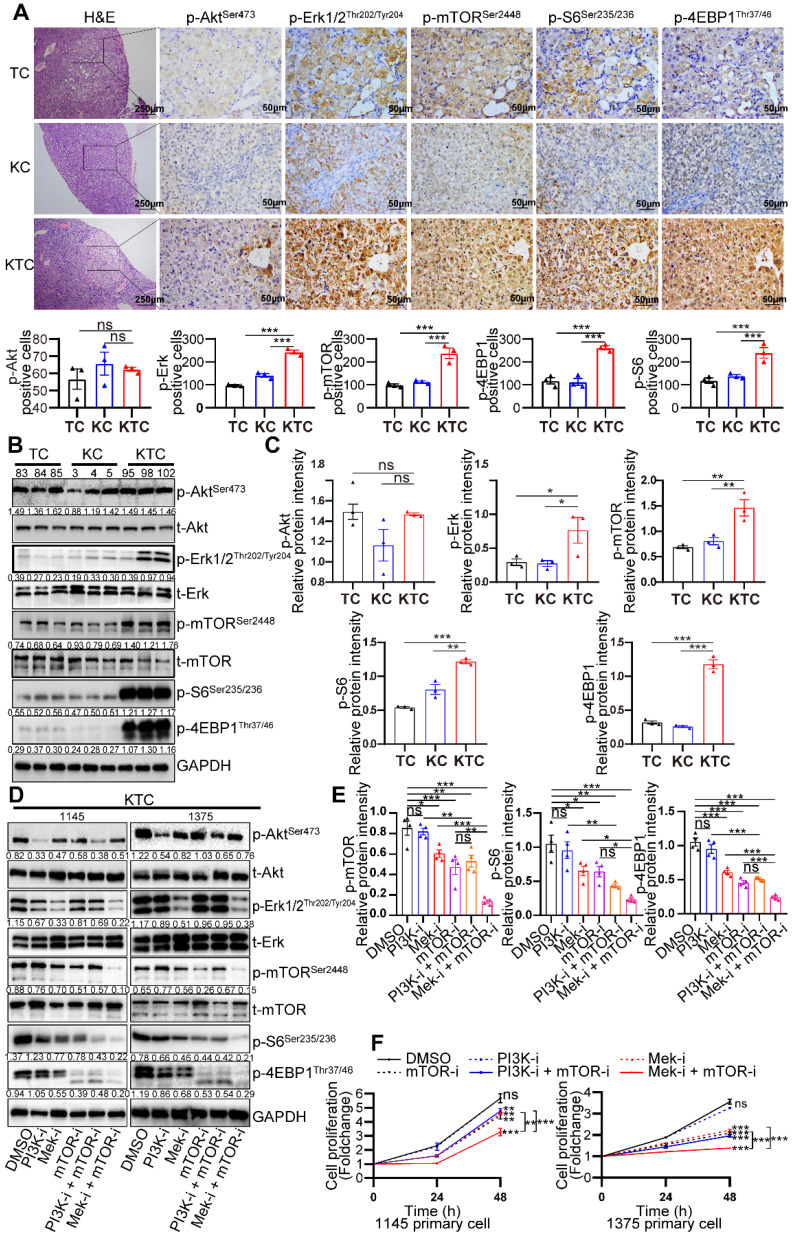
** Mutant Kras activates the Mek/Erk axis, enhancing oncogenic mTOR signaling.** (**A**) Representative consecutive IHC images of Erk/mTOR and Akt/mTOR signals are shown in TC, KC and KTC tumors. Cells with p-Akt^Ser473-^, p-Erk1/2^Thr202/Tyr204^-, p-mTOR^Ser2448^-, p-4EBP1^Thr37/46^- or p-S6^Ser235/236^-positive signals were counted among a total of 500 cells on average from 3 independent tumors derived from 3 mice per group. Images were obtained at 10X or 40X magnification; scale bar, 250 or 50 µm. (**B**) WB showing the phosphorylation levels of p-Akt^Ser473^, p-Erk1/2^Thr202/Tyr204^, p-mTOR^Ser2448^, p-S6^Ser235/236^ and p-4EBP1^Thr37/46^ in TC (#83, 84 and 85), KC (#3, 4 and 5) and KTC (#95, 98 and 102) mice. (**C**) Column representing the statistical analysis of key WB in **B**. (**D**) WB showing the phosphorylation levels of p-Akt^Ser473^, p-Erk1/2^Thr202/Tyr204^, p-mTOR^Ser2448^, p-S6^Ser235/236^ and p-4EBP1^Thr37/46^ in two different KTC primary cell lines (#1145 and 1375) after pharmacological inhibition of Mek (PD98059, 20 µM), PI3K (GDC-0941, 100 nM), mTOR (rapamycin, 100 nM), PI3K + mTOR or Mek + mTOR for 24 h. (**E**) Column representing the statistical analysis of key WB in **D**. (**F**) Proliferation curves for two different KTC primary cell lines (#1145 and 1375) were generated after pharmacological inhibition of PI3K, mTOR, Mek, PI3K + mTOR or Mek + mTOR for 24 or 48 h. Data are represented by the mean ± SEM. **p < 0.01; ***p < 0.001. ns, no significant. One-way ANOVA was used in **A, C and E**; two-way ANOVA was used in **F**.

Further research was conducted to define the interaction of oncogenic Kras^G12D^ mutation and Tsc1 insufficiency driven hyperactivated mTOR, including reciprocal activation/inhibition of upstream and downstream signals. Proteome sequencing data GSE51357 was obtained from GEO. Protein difference analysis (log_2_FC > 0.5, p < 0.05) indicated PTEN/PI3K/Akt signaling and Mek/Erk (MAPK) signaling were hyperactivated after Hras mutation (**Figure S3A**). We found that Akt, Erk and mTOR were consistently hyperactivated after Kras mutation (**Figure S3B**). To validate whether PTEN/PI3K/Akt signaling or Mek/Erk signaling plays a more critical role in the activation of mTOR, liver tumors from TC, KC and KTC mice were analyzed by immunohistochemistry (IHC). As shown in **Figure 2A**, the expression levels of p-Erk1/2^Thr202/Tyr204^ (9/9, 100%), p-mTOR^Ser2448^ (and its downstream targets, p-4EBP1^Thr37/46^ and p-S6^Ser235/236^, 9/9, 100%), but not p-Akt^Ser473^ (1/9, 11.11%), were significantly upregulated in KTC mice compared with TC and KC mice, suggesting that the activation of the Mek/Erk/mTOR axis, but not PI3K/Akt/mTOR axis, is responsible for tumorigenesis in KTC mice. Furthermore, we found that the Mek/Erk pathway in those lung metastases still had high activity and were not influenced by microenvironmental factors in the lung (**Figure S3C**).

Protein was isolated from the liver tumors of TC (#83, 84 and 85), KC (#3, 4 and 5) and KTC mice (#95, 98 and 102). Consistently, p-Erk1/2^Thr202/Tyr204^ and p-mTOR^Ser2448^ but p-Akt^Ser473^ were significantly highly expressed in KTC tumors compared with KC and TC tumors (**Figure 2B-C**). Consistently, reconstitution of Tsc1 in KTC-derived cell lines significantly downregulated the p-Erk1/2^Thr202/Tyr204^ (**Figure S3D**). These findings indicated that mTOR signal activation was necessary for oncogenic Kras to fully activate Erk in tumors. To further substantiate these activation patterns, we investigated the influence of pharmacologically inhibiting Mek (using PD98059), PI3K (using GDC-0941) and/or mTOR (using rapamycin) signaling in two different KTC primary cell lines (#1145 cells and 1375 cells). IC50 values of Rapamycin, GDC-0941 and PD98059 for KTC primary cell lines were shown (**Fig. S3E-F**). Single inhibition of Mek/Erk could significantly reduce the activation of mTOR and its downstream targets p-4EBP1^Thr37/46^ and p-S6^Ser235/236^, and this effect was much stronger than the inhibitory effect of PI3K/Akt, suggesting that mutant Kras promoted Tsc1 insufficiency-driven mTOR activation mostly via Mek/Erk signaling in HCC tumorigenesis (**Figure 2D-E**). In line with these findings, dual inhibition of Mek/Erk and mTOR instead of PI3K/Akt and mTOR strongly reduced p-S6^Ser235/236^ and p-4EBP1^Thr37/46^ levels (**Figure 2D-E**). To further demonstrate that Kras^G12D^ facilitates Tsc1 insufficiency-driven mTOR by activating Mek/Erk signaling, cell proliferation was analyzed in KTC primary cells in the presence or absence of the indicated inhibitors. Consistently, blocking Mek/Erk signals dramatically decreased cell proliferation, whereas inhibition of PI3K/Akt signals weakly affected cell proliferation. Furthermore, dual inhibition of Mek and mTOR completely blocked cell proliferation, which was much stronger than Mek or mTOR single-inhibitor treatment (**Figure 2F**). Taken together, Mek/Erk signals are mainly involved in hepatocarcinogenesis driven by Kras^G12D^ mutation and Tsc1 insufficiency.

### ROS generation is essential for Kras mutation and Tsc1 insufficiency-driven mTOR hyperactivation

**Figure 3:**
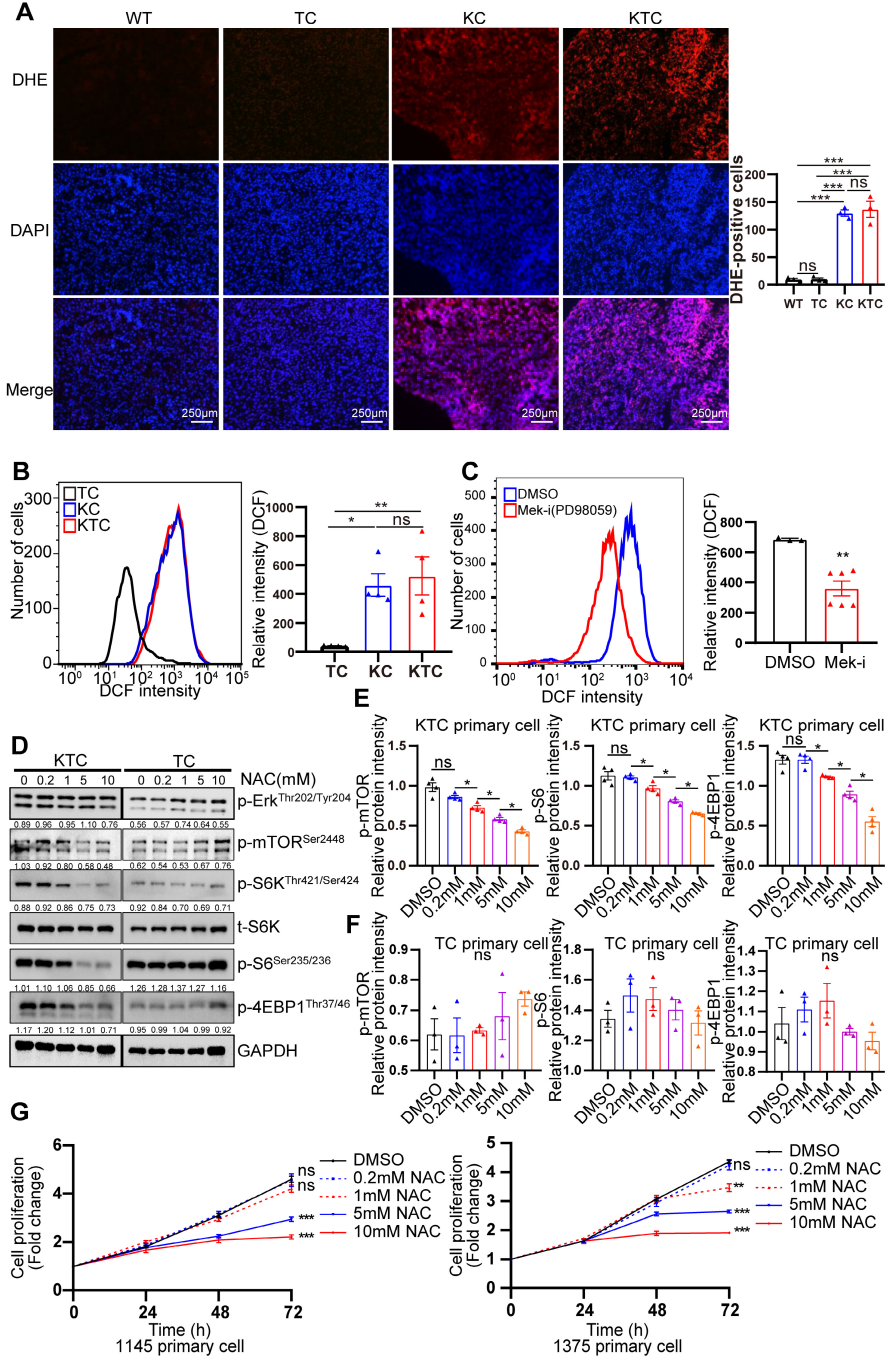
** ROS generation is essential for Kras mutant- and Tsc1 insufficiency-driven mTOR hyperactivation.** (**A**) Representative images of ROS measurement through dye dihydroethidium (DHE)-induced fluorescence in WT, TC, KC and KTC tumors. Cells positive for DHE staining were counted among a total of 500 cells on average from 3 independent tumors derived from 3 mice per group. Images were obtained at 10X magnification; scale bar, 250 µm. (**B**) ROS levels were measured as DCF fluorescence by flow cytometry in TC, KC and KTC tumor cells. n = 3 independent experiments. (**C**) Flow cytometry showed ROS levels (DCF intensity) of KTC primary cells after pharmacological inhibition of Mek (PD98059, 20 µM) for 48 h. n = 6 and 3 independent experiments. (**D**) WB showing the phosphorylation levels of p-Akt^Ser473^, p-Erk1/2^Thr202/Tyr204^, p-mTOR^Ser2448^, p-S6^Ser235/236^ and p-4EBP1^Thr37/46^ in KTC and TC primary cell lines after pharmacological inhibition of ROS (N-acetyl-L-cysteine, NAC) at different concentrations (0, 0.2, 1, 5, 10 mM) for 48 h. (**E-F**) The relative band intensities from WB experiments in **D** were normalized to the level of GAPDH. (**G**) Proliferation curves for two different KTC primary cell lines (#1145 and 1375) were generated after pharmacological inhibition of NAC at different concentrations (0, 0.2, 1, 5, 10 mM) for different time internals. Data are represented by the mean ± SEM. *p < 0.05; **p < 0.01; ***p < 0.001. ns, no significant. One-way ANOVA was used in **A**, **B, E and F**; unpaired Student's t-test was used in **C**; two-way ANOVA was used in **G**.

Mutant Kras activated Mek/Erk/mTOR by reducing the Tsc1/Tsc2 complex in pancreatic ductal adenocarcinoma 34. How mutant Kras facilitates mTOR respond to Tsc1 insufficiency in HCC is unclear. Thus, genetic difference analysis of two Kras mutation cohort GSE53630 and GSE105147 obtained from GEO were conducted. Gene Ontology (GO) analysis of indicated that multiply oxidation-reduction activities and superoxide metabolic process were involved in Kras mutation (**Figure S4A-B**). Due to homeostasis of redox relies on balance between antioxidant proteins and reactive oxygen species (ROS) 35, disruption of redox suggests the unbalance of antioxidant and ROS after Kras mutation. Consistently, Wikipathways enrichment analysis furtherly demonstrated that oxidative stress and redox pathway were activated after Kras mutation (**Figure S4C**). To validate it, the levels of ROS in TC, KC and KTC tumors were detected after dihydroethidium (DHE) staining, showing KC and KTC tumors generated much more ROS than TC tumors or normal liver tissue from WT mice (**Figure 3A**). Moreover, the antioxidant ROS genes *Cat*, *Sod1* and *Sod2* were significantly decreased in KTC and KC tumors compared to WT tissues (**Figure S5A**). Consistently, flow cytometry data also demonstrated that KTC primary cells expressed higher levels of ROS than TC but not KC primary cells (**Figure 3B**). Inhibition of Mek but not mTOR significantly reduced ROS accumulation in KTC primary cells, suggesting that mutant Kras generates ROS in KTC tumors (**Figure 3C, Figure S5B-C**). We next wanted to know whether mutant Kras facilitates Tsc1 insufficiency-driven mTOR activation via ROS generation. KTC primary cells were treated with N-acetyl-L-cysteine (NAC), an ROS scavenger, at different concentrations. We found that NAC significantly inhibited ROS generation and decreased the phosphorylation levels of mTOR, S6K, 4EBP1 and S6 in KTC primary cells, whereas p-Erk1/2^Thr202/Tyr204^ did not change (**Figure 3D-F, S5D**). Furthermore, cell proliferation was analyzed in KTC primary cells in the presence or absence of NAC. Consistently, NAC dramatically decreased KTC primary cell proliferation (**Figure 3G**). Together, ROS generation is essential for mutant Kras- and Tsc1 insufficiency-driven mTOR hyperactivation.

### PEG3 is a biomarker for oncogenic Mek/Erk/ROS/mTOR-driven HCC

**Figure 4:**
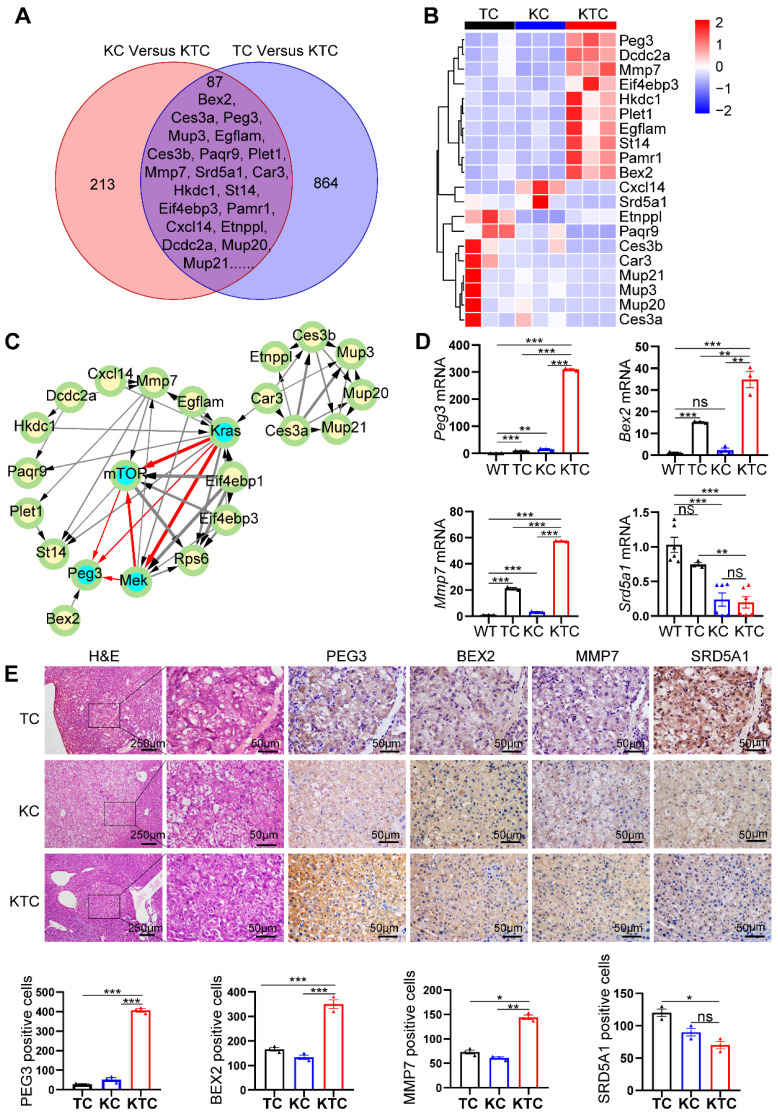
** PEG3 is a biomarker for Mek/Erk/ROS/mTOR-driven HCC.** (**A**) Venn diagram showing significantly changed genes at the mRNA level in liver cancer tissues between KC and KTC mice and between TC and KTC mice. (**B**) A heatmap illustrating the top 20 gene signatures representing the relative gene expression levels in different groups. (**C**) Directed acyclic graph illustrating Protein-protein interaction (PPI) of top 20 significantly changed genes and Kras/Mek/mTOR pathway nodes. (**D**) qPCR showing the relative expression of *Peg3*, *Bex2*, *Mmp7* and *Srd5a1* in different groups. (**E**) IHC demonstrating the expression of BEX2, PEG3, MMP7 and SRD5A1 in different groups. Cells positive for BEX2, PEG3, MMP7 and SRD5A1 signals were counted among a total of 500 cells on average from 3 independent tumors derived from 3 mice per group. Images were obtained at 10X or 40X magnification; scale bar, 250 or 50 µm. Data are represented by the mean ± SEM. *p < 0.05; **p < 0.01; ***p < 0.001; ns, no significant. One-way ANOVA was used in **D** and **E**.

We proposed that Kras^G12D^ mutation and Tsc1 insufficiency cooperate to drive unique gene expression programs in cancer cells to promote HCC progression and metastasis. To survey the genome-wide gene expression at the transcription level in an unbiased manner, we first carefully micro-dissected primary liver tumor nodules in TC, KC and KTC mice. Subsequently, transcriptional profiles among a panel of liver tumor samples from TC, KC and KTC mice were compared. The overlapping genes in these three genotypes were considered potential biomarkers for HCC patients with concurrent Kras mutation and mTOR signaling activation. Using Fragments Per Kilobase Million (FPKM) > 0.5, log_2_FC > 1.5 and a statistical cutoff for FDR < 0.05, 864 genes were found to be differentially expressed between TC and KTC mice, and 213 genes were found between KC and KTC mice, 87 of which were overlapping genes (**Figure 4A**). The top 20 genes with the most significant changes are shown in **Figure 4B-C**, and the rest are shown in **Figure S6A**. To explore the functional relevance of these genes in HCC, we analyzed the survival of HCC patients using the TCGA database. Except for 5 genes that were missed in the TCGA database, the remaining 15 genes were analyzed (**Figure S6B**). Based on the fold change of transcriptional profiles, three up-regulated genes, *Bex2*, *Peg3*, *Mmp7* and one down-regulated gene *Srd5a1* were considered to be most significantly associated with survival in HCC patients.

To further validate the transcriptome data, qPCR, IHC and WB were used to analyze the expression levels of these genes in WT, TC, KC and KTC mice (**Figure 4D-E**, **Figure S7A**). Consistent with the transcriptional profiles, the results indicated that PEG3, BEX2, and MMP7 were significantly increased in KTC tumors and that SRD5A1 was significantly decreased in KTC tumors compared to TC and KC tumors at both mRNA and protein levels. Notably, of these 15 proteins, PEG3 exhibits the highest HR for prognosis risk (**Figure S6B**), which suggests that targeting this molecule may prove beneficial for these particular HCC patients. Moreover, protein-protein interaction (PPI) network analysis also indicates PEG3 has much closer relationship with Kras/Mek/Erk/mTOR signaling than other proteins (**Figure 4C**). Thus, we speculated PEG3 may be a more suitable biomarker for oncogenic Kras/Mek/Erk/ROS/mTOR-driven HCC. Furthermore, IHC showed that PEG3 had higher expression in the tumor sections from KC mice than in the paracancerous sections from KC mice and normal tissues obtained from WT mice (**Figure S7B**). Moreover, we analyzed the relationship between clinically approved well-known HCC biomarkers such as GPC3, AFP, DCP, and PEG3. PEG3 expression was positively associated with GPC3 (Pearson correlation, TCGA: R = 0.35, p < 0.0001; PRIDE-PXD006152: R = 0.38, p < 0.0001) and AFP (Pearson correlation, TCGA: R = 0.46, p < 0.001; PXD006152: R = 0.32, p = 0.0003), but not DCP (Pearson correlation, TCGA: R = 0.18, p = 0.028; PXD006152: R = 0.13, p = 0.15) at both mRNA and protein levels (**Figure S8A-B**). All markers were highly expressed in KTC tumors, but the fold change between KTC and KC tumors for Peg3 (26.17 fold-change) was more significant than the other pre-existing markers (< 4 fold-change) (**Figure S8C**). These findings indicate that PEG3 exhibits superiority over preexisting HCC markers.

To validate whether PEG3 is responsible for the oncogenic Mek/Erk/ROS/mTOR axis, we detected the impacts of mTOR, Mek/Erk, PI3K/Akt, or ROS inhibitors on PEG3 expression. As shown in **Figure S9A-C**, PEG3 expression was significantly reduced by inhibition of Mek, mTOR or ROS but not reduced by inhibition of PI3K in primary KTC cells. Moreover, silencing PEG3 did not change the expression of p-Erk1/2^Thr202/Tyr204^, p-mTOR^Ser2448^, or p-4EBP1^Thr37/46^ or ROS generation (**Figure S9D-F**). Taken together, PEG3 is a downstream target of the Mek/Erk/ROS/mTOR axis.

### PEG3 serves as a novel prognostic predictor in Asian HCC patients with Kras/Erk and mTOR hyperactivation

**Figure 5:**
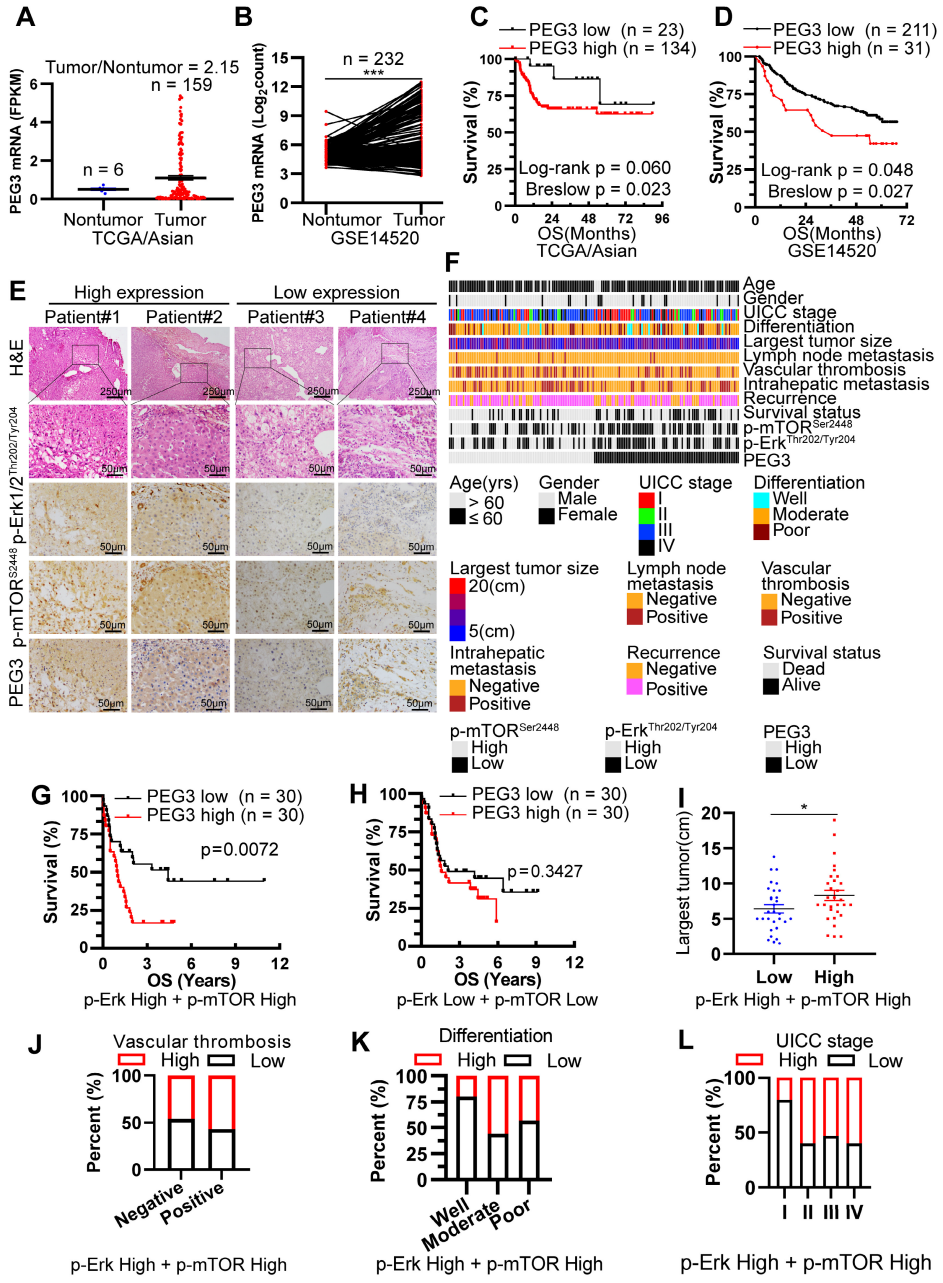
** PEG3 serves as a new poor prognostic marker in HCC patients with Kras/Erk and mTOR hyperactivation.** (**A**) Scatter plots showing the expression level of PEG3 in Asian HCC patients' tumors (n = 159) and adjacent normal tissues (n = 6) (tumor/nontumor tissues = 2.15) obtained from TCGA database. (**B**) Paired scatter plots showing the expression of PEG3 in 232 paired HCC tumors and adjacent nontumor tissues obtained from GSE14520 dataset (paired Student's t-test, p < 0.0001). (**C**) Kaplan-Meier analysis showing the overall survival of Asian HCC patients with high (n = 134) or low (n = 23) expression of PEG3. Data were gotten from TCGA. (**D**) Kaplan-Meier analysis showing the overall survival of HCC patients with high (n = 211) or low (n = 31) expression of PEG3 in GSE14520 dataset. (**E**) Representative images showing the expression levels of p-Erk1/2^Thr202/Tyr204^, p-mTOR^Ser2448^ and PEG3 in HCC samples. p-Erk1/2^Thr202/Tyr204^, p-mTOR^Ser2448^- and PEG3-positive cells were counted among 500 cells. Images were obtained at 10X or 40X magnification; scale bar, 250 or 50 µm. (**F**) A heatmap illustrated different expression of PEG3, p-Erk1/2^Thr202/Tyr204^, p-mTOR^Ser2448^ in each HCC patients. Each column represents a patient sample and rows indicate clinical characteristics and proteins. Relative abundance of the proteins is based on IHC. (**G**) Kaplan-Meier analysis of the overall survival of patients with high (n = 30) or low (n = 30) PEG3 expression in both high activation of p-Erk1/2^Thr202/Tyr204^ and p-mTOR^Ser2448^ HCC samples. (**H**) Kaplan-Meier analysis of the overall survival of patients with high (n = 30) or low (n = 30) PEG3 expression in both low activation of p-Erk1/2^Thr202/Tyr204^ and p-mTOR^Ser2448^ HCC samples. (**I**) Scatter plots of the largest tumor size in patients with high or low PEG3 expression (unpaired Student's t-test, p = 0.044). (**J**-**L**) Distribution of PEG3 according to vascular thrombosis (**J**), tumor differentiation (**K**) or UICC stage (**L**) analyzed by IHC.

Since PEG3 was demonstrated as a biomarker of Mek/Erk/ROS/mTOR-driven hepatocarcinogenesis in response to Kras^G12D^ mutation and Tsc1 insufficiency in murine models, we attempted to determine whether this biomarker was also applicable to HCC patients. Firstly, we analyzed the PEG3 expression level in 355 cases of HCC tumors and 46 cases of adjacent normal tissues obtained from TCGA database. Interestingly, PEG3 was highly expressed in Asian HCC tumors but not in non-Asian's compared with adjacent normal tissues (**Figure 5A**, **S10A**). Due to the limited cases of matched tumors and adjacent normal tissues obtained from TCGA cohort, we furtherly download 232 paired Asian HCC tumors and normal tissues from GEO database (GSE14520) and 124 from PRIDE database (PXD006512) 36. Consistently, PEG3 was higher expression in HCC tumor tissues (paired Student's t-test, GSE14520: p < 0.0001, PXD006512: p = 0.0036) than in non-tumor tissues (**Figure 5B, S10B**). Moreover, high expression of PEG3 was positively associated with poor prognosis of Asian HCC patients rather than non-Asian patients (**Figure 5C-D**,** S10C**, all cutoff points were defined by surv_cutpoint function of R *survminer* package), which also suggests PEG3 as a biomarker in Asian HCC. And then, p-Erk1/2^Thr202/Tyr204^, p-mTOR^Ser2448^ and PEG3 expression levels were analyzed using IHC in 166 HCC samples obtained from surgical resection in our hospital (**Figure 5E-F**). Logistical regression was used to examine the relationship between PEG3 and p-Erk1/2^Thr202/Tyr204^ or p-mTOR^Ser2448^. As shown in **Table S2-S3**, PEG3 expression was positively related to the activation levels of Erk1/2 and mTOR, implying that PEG3 is involved in the Erk/mTOR signaling pathway.

Among all HCC samples, we classified 60 HCC samples with high activation of p-Erk1/2^Thr202/Tyr204^ and p-mTOR^Ser2448^, while 60 with low activation of p-Erk1/2^Thr202/Tyr204^ and p-mTOR^Ser2448^. Kaplan-Meier analysis demonstrated that increased PEG3 expression was positively correlated with poor survival in HCC patients with high activation of p-Erk1/2^Thr202/Tyr204^ and p-mTOR^Ser2448^ (Log-rank test, p = 0.0072; **Figure 5G**), whereas no significant association was found in HCC patients with low activation of p-Erk1/2^Thr202/Tyr204^ and p-mTOR^Ser2448^ (Log-rank test, p = 0.3427, **Figure 5H**). We next examined the relationships between PEG3 protein expression and clinicopathological parameters in HCC patients with high activation of p-Erk1/2^Thr202/Tyr204^ and p-mTOR^Ser2448^. We found that PEG3 expression was positively associated with the largest tumor size (unpaired Student's t-test, p = 0.044), vascular thrombosis, differentiation, and UICC stage (Chi-square test, p = 0.038, **Figure 5I-L, Table 1**). In addition, we also analyzed the relationship between PEG3 mRNA expression and clinicopathologic characteristics in 157 TCGA Asian and 195 non-Asian HCC patients (**Figure S10D, Table S4-S5**). Consistently, PEG3 expression was positively associated with AFP level, UICC stage, UICC tumor stage and pathological grading in Asian HCC patients (**Figure S10E-H, Table S4**), whereas these relationships were not observed in non-Asian patients (**Table S5**). These results indicated that PEG3 expression correlated with HCC disease progression. The prognostic value of PEG3 expression was validated by univariate and multivariate analysis using a Cox proportional hazards model (HR: 2.040; 95% C.I.: 1.034 - 4.023; p = 0.040, **Table S6**).

### PEG3 promotes HCC proliferation and metastasis via its interaction with STAT3 and then activation of STAT3-depedent oncogenic signaling network

**Figure 6:**
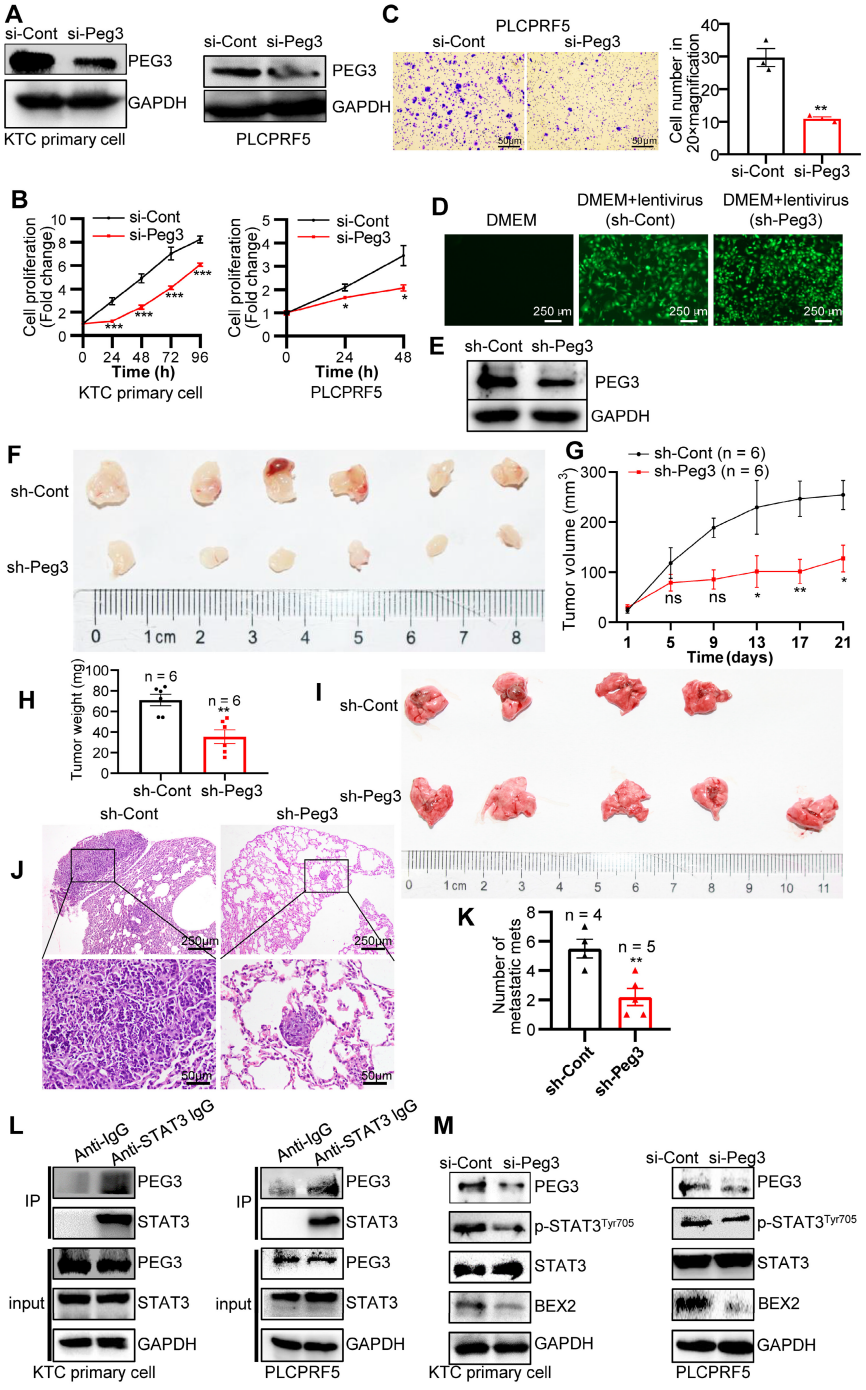
** PEG3 promotes HCC proliferation and metastasis *in vitro* and *in vivo* via STAT3-BEX2 oncogenic signaling network.** (**A**) KTC primary cell, and PLCPRF5 cell line were transfected with a nontargeting siRNA control (si-Cont) or siRNA targeting PEG3 (si-Peg3) for different time intervals. The efficiency of siRNA targeting PEG3 was analyzed by WB after transfection for 48 h. (**B**) Cell proliferation was analyzed by a CCk-8 assay. (**C**) Cell migration was estimated using Transwell assay. **(D-E)** The efficiency of KTC primary cells infected by sh-Peg3 lentivirus was analyzed by fluorescence microscopy (**D**) and WB (**E**). **(F-H)** The subcutaneous xenograft mouse model showing the decrease of tumor volume and weight after knockdown of Peg3. (**I-K**) Tail-vein intravenous injection experiment showing metastatic foci and mets in the lungs after knockdown of Peg3. (**L**) Co-IP experiments showing the interaction between PEG3 and STAT3 in KTC primary cell and PLCPRF5 cell lines. (**M**) WB showing the effect of Peg3 knockdown on p-STAT3^Tyr705^ and its downstream targets BEX2 expression. Data are represented by the mean ± SEM. *p < 0.05; **p < 0.01; ***p < 0.001. Two-way ANOVA was used in **B, G**. Unpaired Student's t-test was used in **C, H, K**.

To further validate the oncogenic functions of PEG3 in HCC, we estimated the effects of PEG3 on cell proliferation or migration in KTC primary cells and PLCPRF5 (Tsc1/Tsc2 mutation) cell line. The results demonstrated that silencing of PEG3 significantly reduced PEG3 protein expression, and inhibited KTC cell proliferation and migration (**Figure 6A-C**). To further validate the oncogenic functions of PEG3 in KTC tumors, we evaluated the effects of PEG3 knockdown on tumor growth and tumor metastasis *in vivo*. Firstly, we established stable Peg3 knockdown KTC primary cells (**Figure 6D-E**). We found that knockdown of Peg3 significantly attenuated mTOR hyperactive HCC tumorigenesis and metastasis using subcutaneous xenograft mouse model and tail-vein intravenous injection *in vivo,* indicated by tumor volume, tumor weight, and lung metastasis (**Figure 6F-K**). Taken together, these results indicate that knockdown of Peg3 significantly block hyperactive mTOR-mediated HCC tumor progress and migration.

Subsequently, we investigated how PEG3 regulates HCC cell proliferation and metastasis. PEG3, an imprinted gene encoding a DNA-binding protein 37, functions as a transcriptional factor or adaptor in interaction with several other proteins in the process of transcriptional regulation. Co-immunoprecipitation experiments indicated that a series of proteins were significantly reduced in PEG3 immunoprecipitates in PEG knockout mouse embryonic fibroblast cells 38, in which signal transducer and activator of transcription (STAT3) was dramatically reduced, suggesting STAT3 may be a PEG3 interacting protein (**Figure S11A-C**). Consistently, we found that endogenous PEG3 interacted with endogenous STAT3 in KTC primary cells and liver cancer PLCPRF5 cell line (**Figure 6L**). Previous studies reported that mTOR up-regulated BEX2 promoted tumorigenesis via STAT3 39. To identify whether PEG3 regulates BEX2 via STAT3, we analyzed the effects of PEG3 knockdown on BEX2 expression. We found that silencing of PEG3 significantly reduced BEX2 expression at both protein and mRNA levels (**Figure 6M, S11D-E**). PEG3 expression was positively associated with BEX2 expression according to TCGA database (Pearson correlation, R = 0.41, p < 0.0001; **Figure S11F**). IHC staining results showed that the activated STAT3 (p-STAT3^Tyr705^) had a high expression level and positively associated with p-ERK1/2^Thr202/Tyr204^ and p-mTOR^Ser2448^ in HCC patient samples with Kras/Erk and mTOR hyperactivation (**Figure S11G**). Furthermore, high expression of STAT3 (p-STAT3^Tyr705^) were positively correlated with the poor prognosis of HCC patients with Kras/Erk and mTOR hyperactivation (**Figure S11H**). Taken together, these results indicate that PEG3 promotes HCC cell proliferation and metastasis through activation STAT3-BEX2 pathway.

### mTOR inhibitors significantly block HCC tumorigenesis and lung metastasis triggered by Kras mutant and Tsc1 insufficiency

**Figure 7:**
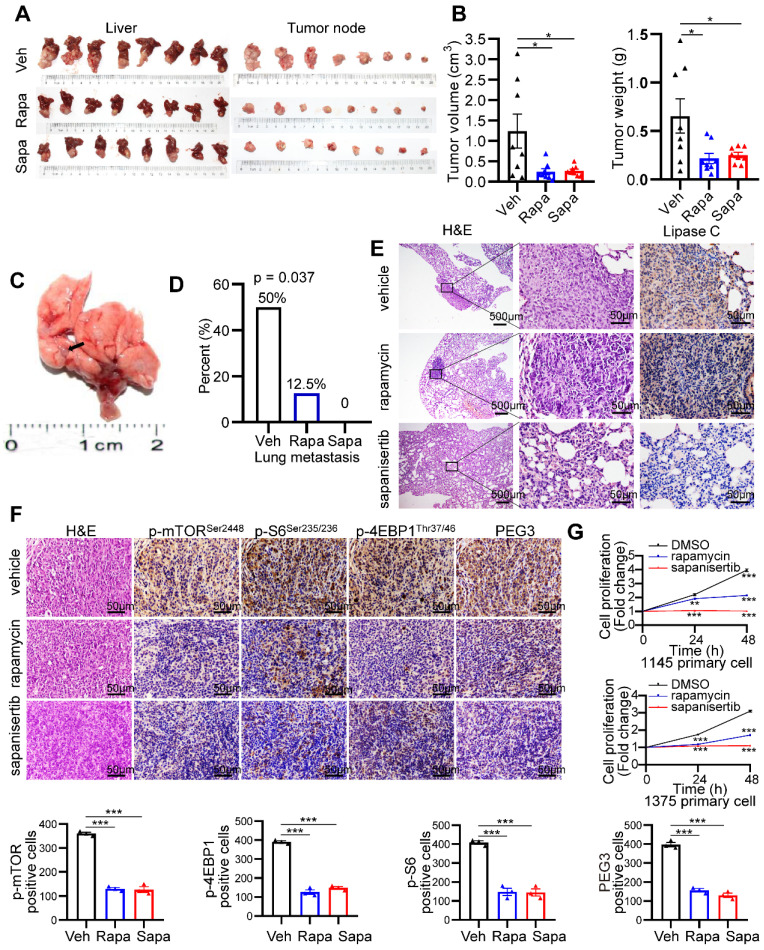
** mTOR inhibitors significantly block hepatocarcinogenesis triggered by Kras mutant and Tsc1 insufficiency.** (**A**) Photos of whole livers with orthotopic tumors (left) and isolated tumors (right) from the vehicle group (Veh; n = 8), rapamycin group (Rapa; n = 8) and sapanisertib group (Sapa; n = 8). (**B**) The tumor volume was calculated using the formula: tumor volume = 3/4 × π × a × b^2^ (a is the longer diameter of the tumor and b is the shorter diameter of the tumor). Tumor volumes and tumor weights were analyzed. (**C**) Macroscopic image of lung metastases was observed in vehicle group. (**D**) The lung metastasis rate was decreased in rapamycin group (1/8) and sapanisertib (0/8), compared with vehicle group (4/8, Pearson Chi-square test, p = 0.037). (**E**) Representative H&E and IHC staining images for Lipase C showing distinct lung metastatic mets expressing Lipase C. Images were obtained at 4X or 40X magnification; scale bar, 500 or 25 µm. (**F**) Representative consecutive IHC images of p-mTOR^Ser2448^, p-4EBP1^Thr37/46^, p-S6^Ser235/236^ and PEG3 in the vehicle group, rapamycin group and sapanisertib group. Cells positive for p-mTOR^Ser2448^, p-4EBP1^Thr37/46^, p-S6^Ser235/236^ and PEG3 signals were counted among a total of 500 cells on average from 3 independent tumors derived from 3 mice per group. Images were obtained at 40X magnification; scale bar, 50 µm. (**G**) Cell proliferation in two KTC primary cell lines (#1375 and 1145) was analyzed using the CCK-8 assay after rapamycin (100 nM) or sapanisertib (1 µM) treatment for different time intervals. *p < 0.05; **p < 0.01; ***p < 0.001. One-way ANOVA was used in **B, F**; Pearson Chi-square test was used in **D**; two-way ANOVA was used in **G**.

Because mTOR activation is involved in Kras^G12D^ mutation and Tscl insufficiency-driven HCC, we investigated the therapeutic effect of the mTOR inhibitors rapamycin and sapanisertib (a new selective inhibitor of mTORC1/2) on hepatocarcinogenesis triggered by mutant Kras and Tscl insufficiency. KTC primary cells were cultured, transplanted to the hypodermis of nude mice, and then retransplanted to the liver of 24 male nude mice. The establishment of an orthotopic HCC model in nude mice and a pharmacological treatment plan are shown in **Figure S12A**. rapamycin (4 mg/kg, i.p., every other day) and sapanisertib (1 mg/kg, i.p. every other day) 40-42 significantly reduced the tumor volume and tumor weight compared to vehicle treatment (**Figure 7A-B**). Interestingly, rapamycin and sapanisertib also decreased the lung metastasis rates in the nude murine model (vehicle: 4/8, 50%; rapamycin: 1/8, 12.5%; sapanisertib: 0/8, 0%. Pearson Chi-square test, p = 0.037, **Figure 7C-E**).

To confirm the inactivation of the mTOR signaling pathway after rapamycin and sapanisertib treatment, IHC and WB for mTOR and its downstream targets were performed. The KTC tumors were p-mTOR^Ser2448^-, p-S6^Ser235/236^-, p-4EBP1^Thr37/46^-, as well as PEG3- positive in the vehicle treatment groups, while these signals were dramatically reduced in the rapamycin and sapanisertib treatment groups (**Figure 7F, S12B**). Consistently, two distinct primary cell lines were significantly susceptible to the anticancer effect of rapamycin or sapanisertib treatment (**Figure 7G**). In addition, the expression of Mek/Erk/mTOR signaling and its downstream targets were also decreased in the lung metastatic mets of nude mice treated with rapamycin and sapanisertib treatment groups compared with those treated with vehicle (**Figure S12C**).

**Figure 8:**
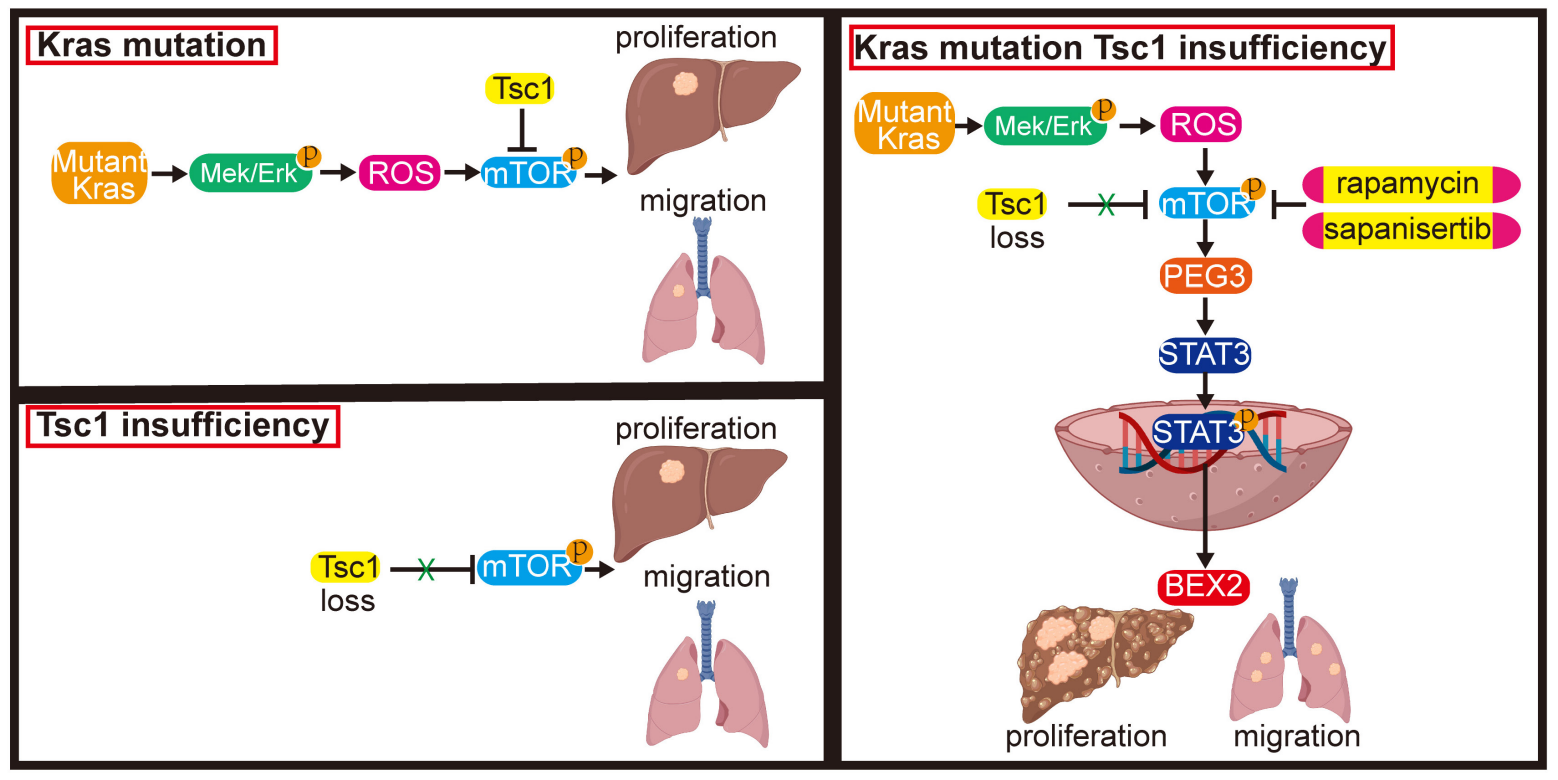
** Model of the role of Kras^G12D^ in regulation of Tsc1 insufficiency-induced HCC tumorigenesis and metastasis.** Under Tsc1 insufficiency conditions, mTOR is weakly activated and induces a long period of liver tumorigenesis. Under Kras mutant conditions, Kras activation initiates the Mek/Erk signaling pathway to generate high levels of ROS, which activate mTOR to induce a long period of liver tumorigenesis. Upon Tsc1 insufficiency and Kras mutant, Tsc1 insufficiency further promotes Kras-Mek-Erk-ROS-mediated mTOR activation, which upregulates PEG3 expression and promotes its interaction with STAT3. Activated STAT3 causes transcriptional activation of BEX2, leading to an accelerated liver tumorigenesis and lung metastasis with a shortened latent period and increased incidence. Targeting of mTOR (e.g., rapamycin and sapanisertib) could significantly inhibit HCC tumorigenesis and lung metastasis in HCC patients with Kras mutant and Tsc1 insufficiency.

These results indicate that inhibition of mTOR represents a potential therapeutic strategy for an aggressive subset of HCC with Kras mutant and Tsc1 insufficiency. Taken together, Kras mutant enhances Tsc1 insufficiency-mediated mTOR activation by Mek/Erk pathway via ROS. Hyperactivated mTOR then upregulates PEG3 expression and promotes its interaction with STAT3, resulting in STAT3-dependent HCC tumorigenesis and metastasis (**Figure 8**).

## Discussion

The rising prevalence of HCC represents a major challenge to global health. The high recurrence rate and frequent metastasis of patients with HCC are consequences of insufficient knowledge of the molecular mechanisms underlying HCC pathogenesis 2. The pathogenesis of HCC is quite complicated and involves multiple molecular pathways with overlapping, complementary or opposing effects.

As frequently deregulated mTOR signaling significantly contributes to HCC development and progression, targeting the mTOR pathway seems to be a promising strategy for HCC treatment 43. Preclinical studies have demonstrated the effectiveness of the first generation of mTOR inhibitors rapamycin derivatives in HCC growth inhibition 44, 45, whereas they had very limited efficacy against advanced HCC in clinical trials 7, 46. Second-generation pan-mTOR inhibitors, such as sapanisertib (MLN0128) and dactolisib (NVP-BEZ235), which predominantly function to inhibit mTOR kinase catalytic activity, are more efficacious 47. However, the results from several clinical trials are inconsistent 7, 48, 49. Due to HCC is a highly heterogeneous disease with a variety of deregulated motecular signaling pathways, therefore, identifying reliable biomarkers of those special genetic settings is critical to facilitate the selection of HCC patients who might benefit from mTOR inhibition.

In the current study, we focus on Kras mutation and hyperactivated mTOR signaling driven by Tsc1 insufficiency, both of which are frequently activated in HCC 50. We firstly speculated whether a functional link exists between Kras mutation and hyperactivated mTOR. Thus, transgenic murine models and *in vitro* signaling analyses were adopted. We found that oncogenic Kras^G12D^ mutation facilitated activation of Mek/Erk-mediated Tsc1 insufficiency-driven mTOR signaling via ROS, which promoted the development and metastasis of HCC.

PEG3 was previously reported as tumor suppressor in various cancers, including lung cancer and breast cancer 23. However, the function of PEG3 in HCC remains unclear. In this study, we found that (1) PEG3 was significantly increased in HCC tissue with high activation of p-Erk1/2^Thr202/Tyr204^ and p-mTOR^Ser2448^ compared with adjacent noncancerous liver tissue; (2) PEG3 expression was also increased in Kras mutation liver tissue compared with wild-type liver tissue; (3) Increased PEG3 expression was positively associated with poor survival in HCC patients with high activation of p-Erk1/2^Thr202/Tyr204^ and p-mTOR^Ser2448^ rather than low activation of p-Erk1/2^Thr202/Tyr204^ and p-mTOR^Ser2448^; and (4) PEG3 was positively associated with cell proliferation in primary liver cells. (5) PEG3 expression was positively associated with p-Erk1/2^Thr202/Tyr204^ and p-mTOR^Ser2448^ expression. (6) PEG3 directly bound to STAT3 and increased its transcription activity, leading tumor proliferation and metastasis. These results indicate that PEG3 functions as oncogene in HCC and is responsible for Kras/Erk and mTOR signaling-triggered HCC tumorigenesis. In clinical samples, PEG3 expression was positively corelated with p-Erk1/2^Thr202/Tyr204^ and p-mTOR^Ser2448^, implying that PEG3 is involved in the Erk/mTOR signaling pathway and can be used as a special prognostic marker for HCC patients with hyperactive Erk and mTOR. Furthermore, PEG3 is significantly associated with UICC stage. Due to limited HCC cases, the p value is significant but not strong (p = 0.038). However, the underlying mechanisms by which oncogenic Ras/Mek/Erk/mTOR axis regulates PEG3 expression is not well understood and needs to be further investigated.

Oxidative stress, which is mediated by cellular increases in ROS, is considered a bona fide tumor promoter, contributing to the initiation and progression of HCC 51, 52. ROS levels increase during the progression from early to advanced HCC 53. Elevated ROS levels could induce Akt activity-mediated upregulation of TERT and telomere maintenance or telomere elongation, thereby resulting in enhanced survival of malignant HCC cells 54. In this study, we found that KTC mice had a higher ROS level than TC or wild-type mice, which is associated with a greater tumor-forming ability. Meanwhile, an ROS scavenger attenuated the hyperactivation of the mTOR pathway in Tsc1 insufficient and Kras-mutated tumor cells. The increased levels of ROS in this system are responsible for enhanced hepatocarcinogenesis through the activation of the mTOR pathway. Oncogenic Kras induced ROS generation through activation of NADPH oxidase1, which is a critical regulator of Kras-induced cellular transformation 55. In myeloid leukemia, oncogenic Kras leads to RAC1 activation, causing NADPH activation and resulting in ROS production, which in turn activates inflammation-related effects 56. Moreover, Kras expression promoted ROS generation through cyclooxygenase (COX)-2, causing DNA damage and malignant transformation in lung epithelial cells. However, the mechanisms that regulate mTOR hyperactivation during Kras/Mek/Erk-induced ROS generation and how ROS promote tumorigenesis still need to be fully understood.

## Conclusions

In conclusion, our study revealed that 1) Kras mutant facilitates mTOR activation via ROS accumulation, leading to HCC tumorigenesis and metastasis; 2) dual inhibition of mTOR and Mek effectively decreases mTOR (hyper)activity; 3) the oncogenic activity of the Ras/Mek/Erk/ROS/mTOR axis relies on PEG3, which binds STAT3 and promotes its transcriptional activity; 4) PEG3 acts as oncogene in HCC and is negatively associated with prognosis in HCC patients with Kras/Erk and mTOR hyperactivation; 5) Targeting mTOR would be an effective strategy for treating HCC patients with Kras/Erk and mTOR hyperactivation.

## Supplementary Material

Supplementary materials and methods, figures, and tables.

## Figures and Tables

**Table 1:** Relationship between PEG3 protein expression and clinicopathologic characteristics in 60 HCC patients with high expression of p-Erk and p-mTOR

Characteristics	N.O. patients (%)	PEG3 expression level		p value
		low	high	
**Gender**				
Male	57(0.95)	29	28	0.554
Female	3(0.05)	1	2	
**Age (years)**				
≤ 60	53(0.88)	27	26	0.688
> 60	7(0.12)	3	4	
**UICC stage**				
I	10(0.17)	8	2	0.038
II + III + IV	50(0.83)	22	28	
**Lymph node Metastasis**				
Negative	57(0.95)	29	28	0.554
Positive	3(0.05)	1	2	
**Largest tumor size**				
< 10cm	13(0.22)	9	4	0.136
≥ 10cm	47(0.78)	21	26	
**Differentiation**				
Well	5(0.08)	4	1	0.161
Middle + Poor	55(0.92)	26	29	
**Vascular thrombosis**				
Negative	37(0.62)	20	17	0.426
Positive	23(0.38)	10	13	
**Recurrence**				
Negative	18(0.3)	10	8	0.573
Positive	42(0.7)	20	22	
**Intrahepatic metastasis**				
Negative	37(0.62)	18	19	0.791
Positive	23(0.38)	12	11	

PEG3, paternally expressed 3. Statistical analyses were carried out using the Pearson Chi-square test. *p ≤ 0.05 was considered statistically significant.

## Abbreviations

DHE: dihydroethidium
FPKM: Fragments Per Kilobase Million
GEO: Gene Expression Omnibus
GO: Gene Ontology
HCC: hepatocellular carcinoma
HR: Hazard ratios
IHC: immunohistochemistry
mTOR: mammalian target of rapamycin
NAC: N-acetyl-L-cysteine
PPI: protein-protein interaction
PRIDE: Proteomics Identifications
ROS: reactive oxygen species
PEG3: paternally expressed 3
Rapa: rapamycin
Sapa: sapanisertib
STAT3: signal transducer and activator of transcription 3
TCGA: The Cancer Genome Atlas
Tsc1: tuberous sclerosis complex 1
