# Supplementary material for: Mutant Kras and mTOR crosstalk drives hepatocellular carcinoma development via PEG3/STAT3/BEX2 signaling
Source: Theranostics. 2022 Nov 16;12(18):7903–19. doi: 10.7150/thno.76873 (PMC9706580; doi:10.7150/thno.76873)
Supplement: Supplementary file 1 — Supplementary materials and methods, figures, and tables. [file thnov12p7903s1.pdf]

Mutant Kras and mTOR crosstalk drives hepatocellular carcinoma development via PEG3/STAT3/BEX2 signaling

Supplementary materials

Reagents and antibodies

Fetal bovine serum (10270), Dulbecco's modified Eagle's medium (C11995500BT) and Opti-MEM (31985-070) were purchased from Gibco. Anti-mTOR^{Ser2448} for WB (#5536), anti-mTOR (#2983S), anti-p-Erk$^{Thr202/Tyr204}$ (#4376), anti-Erk (#4695), anti-Akt^{Ser473} (#4060), anti-Akt (#4691), anti-p-S6$^{Ser235/236}$ (#2211), anti-p-4EBP1$^{Thr37/46}$ (#2855), p-S6K$^{Thr421/Ser424}$ (#9204S), S6K (#9202), anti-Ccnb1 (#12231)，STAT3 (#9139) and p-STAT3^{Tyr705} (#9145) antibodies were purchased from Cell Signaling Technology. Anti-CCNB2 (21644-1-AP), anti-MMP7 (10374-2-AP), anti-SRD5A1 (26001-1-AP) and anti-Lipase C (21133-1-AP) antibodies were purchased from Proteintech. Anti-BEX2 (FNab00884) antibodies were purchased from FineTest, and anti-Peg3 (OAAJ11874) antibodies were purchased from Aviva. Rapamycin (S1039), sapanisertib (S2811), GDC-0941 (S1065), and PD98059 (S1177) were purchased from Selleck. Dihydroethidium (D11347) was purchased from Thermo Fisher. Anti-mTOR^{Ser2448} (SAB4504476) for IHC, DCFHDA (D6883), and N-acetyl-L-cysteine (A7250) were purchased from Sigma. A CCK-8 kit (CK04) was purchased from Dojindo Molecular Technologies.

Supplementary patients and samples

166 cases of hepatocellular carcinoma (HCC) patients were from Southwest Hospital. 355 HCC specimens and 46 adjacent nontumor specimens were obtain from the cancer genomic atlas (TCGA). In the 355 HCC specimens, of which 157 were from Asian patients and 196 from non-Asian patients. From Gene Expression Omnibus (GEO) database, we downloaded 242 cases of Asian HCC patients from GSE14520 dataset, in which 232 cases had a matched tumors and adjacent normal tissues. From Proteomics Identifications (PRIDE) database, we download 124 paired HCC samples and adjacent normal tissues.

Primary cell isolation and culture

Primary cells were isolated as described in our previous publication [1]. Briefly, the mice were anesthetized, disinfected and immobilized, and then laparotomy was performed to expose the liver and inferior vena cava (IVC). The IVC was cannulated with a catheter connected to a perfusion line and perfused with an EGTA solution, pronase solution and collagenase solution. After the liver was perfused and digested in situ, digested and centrifuged in vitro, primary liver cells were isolated successfully. Then, liver primary cells were cultured in DMEM containing 20% FBS and 2% penicillin/streptomycin.

Cell line culture

Liver cancer PLCPRF5 (CRL-8024) cell line was obtained from ATCC. All passed tests of cell line quality control methods (e.g., morphology, isoenzymes, and mycoplasma). All cells were maintained using Dulbecco’s modified Eagle’s medium (DMEM) with 10% fetal bovine serum (FBS), and cultured at 37℃ with 5% CO_2 in a

humidified incubator.

Orthotopic tumorigenesis

First, primary liver cells were successfully isolated from $Kras^{G12D}$;$Tsc1^{fl/fl}$;Alb-Cre mice. Then, 1×10^7 cells were suspended in 100 μl PBS/Matrigel (1:1) and transplanted to the hypodermis of 6-week-old male BALB/c-nude mice (GemPharmatech, Chengdu, China), which were first anesthetized by isoflurane. After two weeks, the nude mice were killed with an excessive anesthetic dose, and the tumor tissue was removed subcutaneously. The tumor was cut into 1 mm^3 pieces and retransplanted into the livers of 32 six-week-old male nude mice that were anesthetized and underwent laparotomy. All mice were were randomly divided into three groups. Thus, orthotopic tumorigenesis was finished [2]. One day after orthotopic tumorigenesis, rapamycin and sapanisertib treatment assays were conducted. Rapamycin and sapanisertib were reconstituted in ethanol at 20 mg/ml and 5 mg/ml, respectively, and then diluted in 5% Tween-80 and 5% PEG-400 to working concentrations of 4 mg/kg and 1 mg/kg, respectively. Mice were treated with rapamycin (4 mg/kg, i.p.) and sapanisertib (1 mg/kg, i.p.) every other day for 3 weeks [3-5]. Finally, nude mice were killed with an excessive anesthetic dose, and tumor volumes and tumor weights were evaluated. Experimenters conducting murine model construction and drug treatment and Experimenters conducting mouse death and data collection were two different groups of experimentalists and followed the blinding principle. All experiments were approved by the Institutional Animal Care and Use Committees of Army Medical University.

Lentivirus infection

KTC primary cells were successfully infected by sh-Cont or sh-Peg3 lentivirus according to the manufacturer's specification. 2×10^7 lentivirus and 100 µl HiTranG A were added on KTC primary cells. After 24 h infection, medium was replaced with complete medium. At 3 days after infection, the transfection efficiency was analyzed by fluorescence microscopy and Western blot.

HCC xenografts mouse model and lung metastasis model

For HCC xenografts model, infected KTC primary cells suspension (5×10^7 cells/ml, in 100 µl 1:1 PBS/Matrigel) were injected subcutaneously into the left flanks of 6-week-old male BALB/c-nude mice. Tumor size was recorded every 4 days. Tumor volume was calculated with the formula of $0.5 \times \text{length} \times \text{width}^2$ [6]. At 3 weeks after injection, the nude mice were euthanized. The tumors were imaged. The tumors were weighted. For lung metastasis model, the infected KTC primary cells (2×10^7 cells/ml, in 100 µl PBS) were injected into 6-week-old male BALB/c-nude mice via tail vein. After 6 weeks injection, the nude mice were euthanized and lung tissues were collected.

Small interfering RNA (SiRNA) transfection

The siRNA sequences for Peg3 and the negative control were synthesized by GenePharma Co. (Shanghai, China). The sequence for mouse si-Peg3 was as follows: 5'- ACTGTACGAATGCAAAGAT -3'. The sequence for mouse si-Cont was as follows: 5'-UUCUCCGAACGUGUCACGUTT -3'. The sequence for human si-Peg3 was as follows: 5'- GAGUGUUGUGUUCAUCUAAUC -3'. The sequence for human

si-Cont was as follows: 5’- UCUACGAGGCACGAGACUU -3’. First, siRNA was mixed in Opti-Medium at a 90 nM concentration with Lipofectamine 2000. After reacting for 20 minutes at room temperature, the mix was added to the cells for the indicated time intervals.

H&E staining

Liver tissues were fixd in 10% formalin for at least 24 h and embedded in paraffin. Then, 4-μm-thick tissue sections were cut and dewaxed, rehydrated and stained with hematoxylin for 30 seconds, followed by eosin staining for 20 seconds. The slides were naturally dried and covered with neutral gum. Images were acquired with a light microscope (BX41, Olympus).

Immunohistochemistry (IHC)

Briefly, tissue slides were dried at 60 °C for 2 h, deparaffinized with xylene and rehydrated through a graded alcohol series. Endogenous peroxidase activity was blocked by incubation in 3% hydrogen peroxide for 30 minutes. Subsequently, antigen retrieval was carried out by immersing the slides in 10 mM sodium citrate buffer (pH 6.0) and maintaining them at a boiling temperature for 10 minutes. After cooling to room temperature, the slides were blocked in 1% blocking serum for 30 minutes and then incubated with the primary antibody at 4 °C in a humidified chamber. Subsequently, the sections were incubated with biotinylated secondary antibody and then streptavidin-labeled horseradish peroxidase. Finally, the signal was developed with diaminobenzidine, and the slides were counterstained with hematoxylin. The staining intensity was determined using the Spot Denso function of AlphaEaseFC

software. Positively stained cells were counted among a total of 500 cells on average from 3 independent tumors/inflammation tissues derived from 3 mice per group.

Sirius red staining

Tissue samples were fixed, embedded, dewaxed and rehydrated for H&E staining. Then, the tissue sections were stained with Sirius red solution for approximately 30 minutes. The slides were naturally dried in air at room temperature and covered with neutral gum. Images were acquired using the light microscope.

Western blot analysis (WB)

Proteins were extracted from cultured cells or tissues using PIPA buffer containing protease inhibitor cocktails. The lysate was centrifuged at 12,000 rpm for 20 minutes at 4 °C. The supernatant was collected, and the protein concentration was determined using a BCA assay. For western blotting, protein samples were boiled with SDS–PAGE loading dye and loaded at ~20 μg into each lane and separated by SDS–PAGE gel. For immunoblots, the nitrocellulose membrane carrying the transferred proteins was blocked with 5% BSA for 1 h at room temperature with constant shaking and then blotted with primary antibodies at 4 °C overnight. After washing three times for 15 minutes each in TBST buffer, immunodetection was accomplished using a horseradish peroxidase-conjugated secondary antibody and the Super-Signal West Pico Chemiluminescent Substrate. The relative band intensities from western blot experiments were normalized to the level of GAPDH. The relative band intensities were measured by a Quantity One software (V4.6.6) and added below the bands.

Co-immunoprecipitation (Co-IP) experiment

Proteins were extracted from HCC cells using 1 ml IP Lysis buffer. 200 μl was reserved for input detection. The remaining was centrifuged at 4 ˚C for 15 minutes at 12,000xg to remove cell debris. The cleared lysate was added with 20 μl of the protein A/G agarose bead (Santa Cruz, sc-2003) for 5 minutes at 4 ˚C with gentle agitation. And then, the sample was centrifuged at 2500 rpm for 5 minutes to remove the protein A/G agarose bead. Next, another 60 μl of the protein A/G agarose bead and 5 μl anti-STAT3 antibody were added to the sample again and incubated overnight at 4˚C with gentle agitation. Tomorrow, the sample was centrifuged at 2500 rpm for 5 minutes to remove the supernatants, and the remaining beads were washed three times with the lysis buffer. The proteins were eluted from the beads with 50μl 1x loading buffer for 5 minutes at 100℃. The immunoprecipitated proteins were analyzed through WB as described before.

RNA isolation and reverse transcription-polymerase chain reaction (RT–PCR)

Total RNA was isolated by RNAiso Plus (Takara; 9180). RT–PCR was performed with the PrimeScript™ RT reagent kit (Takara; RR037A) according to the manufacturer’s instructions. Quantitative real-time PCR (qPCR) was performed using TB Green™ Premix Ex Taq™ (Takara; RR420A). The results were obtained with CFX96TM Real-time System 3.1 software (Applied Bio–Rad). *Gapdh* was used as an internal control. Specific primer sequences are listed in **Table S7**.

Cell growth assay

For the cell viability assay, the cells were seeded in 96-well plates at a density of

8,000 cells (for primary cell) or 2,000 cells (for cell lines) per well overnight, treated with the respective agents for the indicated duration and then exposed to 10 μl of CCK-8 solution per well for 1 h at 37 °C. Absorbance was measured at 570 nm with a microplate reader.

Cell migration assay

The cells were firstly seeded in 6-well plates at a density 2×10^5 cells per well overnight. Transfection with the si-Peg3 and nontargeting siRNA for 24 h. Cells were digested by trypsin and plated into Transwell plate without FBS overnight. Finally, cells were fixed by 4% paraformaldehyde for 10 minutes, and then stained by 0.1% crystal violet for 30 minutes. Images were acquired using the light microscope.

ROS analysis using DCFHDA staining

First, the dated medium was discarded, and the cells were washed with sterile PBS 3 times. Then, the cells were cultured with fresh medium containing 10 nM DCFHDA for 20 minutes at 37 °C in the dark. Next, the fresh medium was discarded again, followed by washing 3 times with sterile PBS. Images of ROS were observed with a fluorescence microscope with an equal exposure setting in each group. The fluorescence intensity was analyzed using Image-Pro Plus software in every group, and the production of ROS was indicated. Additionally, the cells were digested and collected. Flow cytometry was performed to evaluate superoxide production in cells.

ROS analysis using dihydroethidium (DHE) staining

Frozen sections of liver tissue were incubated with DHE at 37 °C for 40 minutes and then fixed with paraformaldehyde for 10 minutes. Next, DAPI staining solution

was used to stain the nucleus for 10 minutes. Finally, images were captured using a fluorescence microscope at an excitation wavelength of 490 nm and an emission wavelength of 590 nm. The exposure time used for image acquisition of all sections was 30 ms. The intensity of DHE fluorescence was quantified by an analysis system.

High-throughput mRNA sequencing

Liver tumor nodules were obtained at 280 days from Tsc1$^{fl/fl}$; Alb-Cre (TC); Kras^{G12D};Alb-Cre (KC); and Tsc1$^{fl/fl}$; Kras^{G12D};Alb-Cre mice (KTC). Total RNA was carefully prepared, and RNA-seq analysis was performed (Genergy Bio, Shanghai, China). RNA was extracted and inspected, and a library was constructed. Then, bridge PCR amplification was performed on the cBot instrument to generate clusters, and 2 × 150 sequencing mode was used for sequencing on the Illumina sequencing platform. Briefly, after a series of data analyses, the final sequencing results were obtained.

Limma package was used to analyze the differential gene expression between TC/KC and KTC mice [7]. The length data of whole genes were obtained from GENCODE (https://www.gencodegenes.org/). Thus, normalized read counts and Fragments Per Kilobase Million (FPKM), were calculated. p values were adjusted using the Benjamini–Hochberg procedure for multi-testing, and these false discovery rate (FDR) were computed [8]. Thus, these arguments were used to define the most significant change gene in KTC mice compared with TC or KC mice.

Bioinformatics analysis

The human HCCs were based on data generated by The Cancer Genome Atlas (TCGA) database. The clinical characteristics were obtained from Liver

Hepatocellular Carcinoma (TCGA, Firehose Legacy) in cBioPortal (http://www.cbioportal.org/). The FPKM data were obtain from the human protein atlas (https://www.proteinatlas.org/) and UCSC xena (https://xenabrowser.net/datapages/?cohort).

Proteome sequencing data GSE51357 and RNA sequencing data GSE105147, GSE53630, GSE14520 were obtained from Gene Expression Omnibus (GEO) database (https://www.ncbi.nlm.nih.gov/geo/). Expression difference analysis was used by *limma* package [7], as described above. Gene Ontology (GO) and Wikipathways enrichment and clustering analysis were carried out using DAVID Bioinformatics Resources (DAVID 6.8, https://david.ncifcrf.gov/) [9, 10]. The significant results were extracted and plotted with R [11]. Proteome data PXD006512 was obtained from PRIDE (Proteomics Identifications) database (https://www.ebi.ac.uk/pride/archive/) [12] .

A co-immunoprecipitation experiment result of Peg3 knockout and wild type in mouse embryonic fibroblast cells using tandem mass spectrometry was obtained from an online literature's supplementary materials [13]. Protein expression difference analysis was also carried out using R *limma* package, followed volcano plot was drawn with R. Protein interaction networks were retrieved from STRING v.11.5 database (https://cn.string-db.org/) [14, 15] and visualized using Cytoscape v.3.9.1 [16].

Supplementary figures

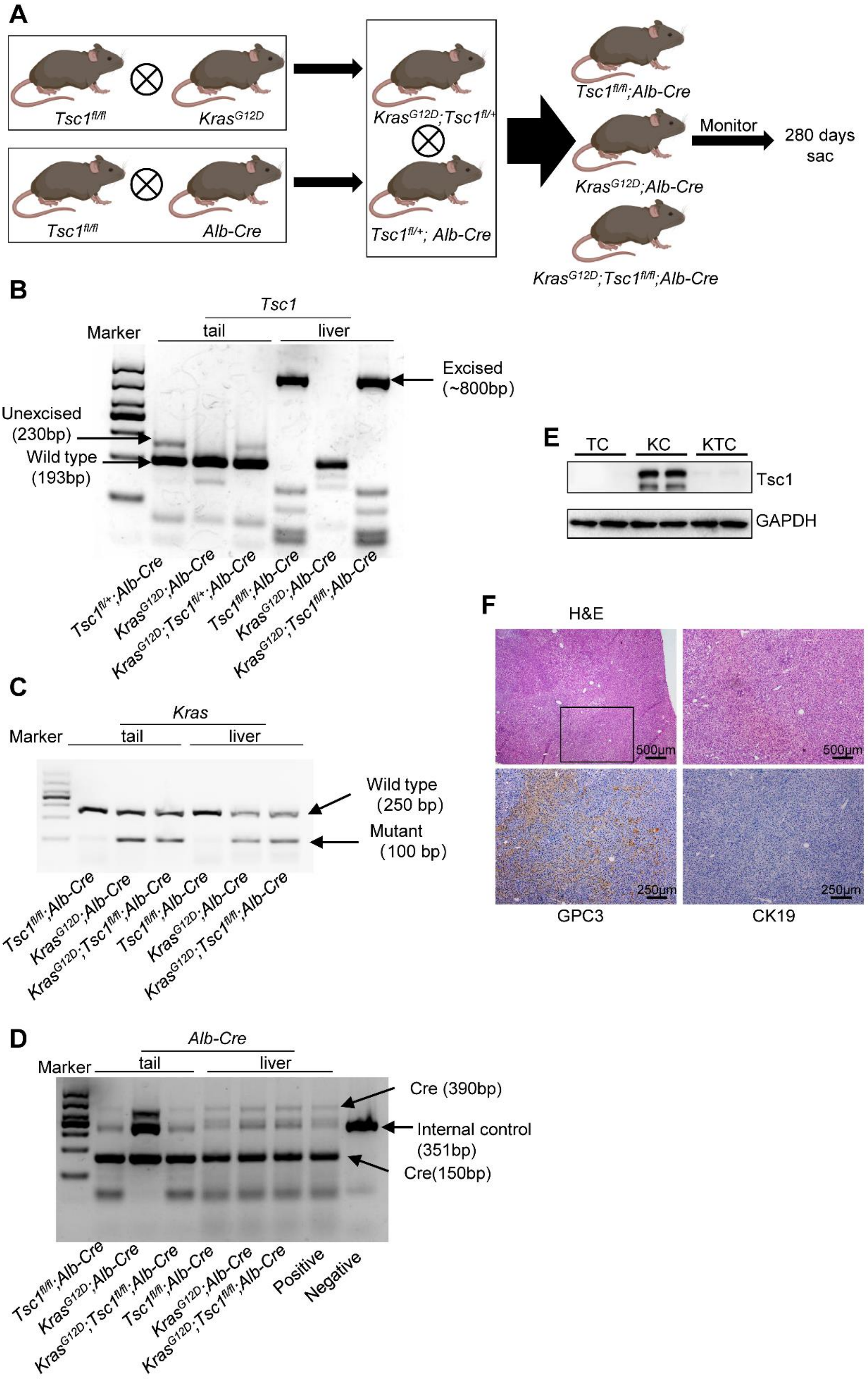

Figure S1. PCR genotyping illustrating *Kras*^{G12D};*Tsc1*$^{fl/fl}$ alleles and *Alb-Cre* in

offspring. (**A**) *Kras*^{G12D}*;Tsc1*$^{fl/fl}$*;Alb-Cre* were generated by intercrossing Kras^{G12D} mice with Tsc1 $^{fl/fl}$ mice, followed by crossing with *Alb-Cre* mice. Schematic diagram showing all *Tsc1*$^{fl/fl}$*;Alb-Cre*, *Kras*^{G12D}*;Alb-Cre* and *Kras*^{G12D}*;Tsc1*$^{fl/fl}$*;Alb-Cr*e mice were monitored 280 days followed by sacrificed. (**B-D**) DNA was isolated from tail and liver tissue from the same mice with the indicated genotype and subjected to PCR genotyping for *Tsc1* deletion (**B**), *Kras* ^{G12D} mutation (**C**) and *Alb-Cre* (**D**). (**E**) WB showing the loss of Tsc1 in TC and KTC mice. (**F**) Representative H&E and IHC staining images for GPC3 and CK19 sections from KC, TC and KTC tumors. Images were obtained at 4X or 10X magnification; scale bar, 500 or 250 µm. The black box indicates the area used for the high-magnification images.

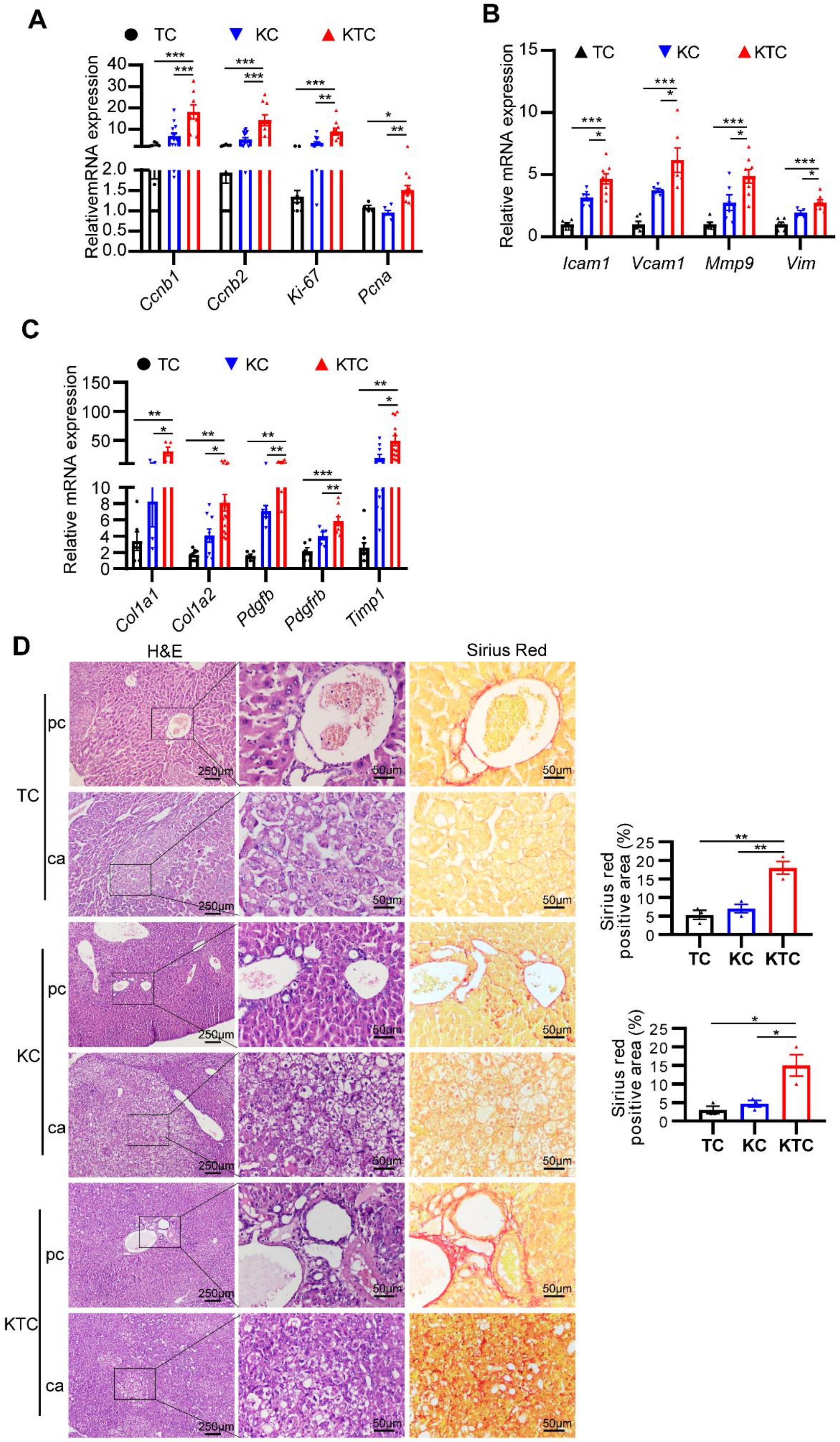

Figure S2. Mutated Kras facilitates Tsc1 insufficiency-driven liver fibrosis. qPCR

showing the proliferation markers *Ccnb1*, *Ccnb2*, *Ki-67* and *Pcna* (**A**), the metastasis markers *Icam1*, *Vcam1*, *Mmp9* and *Vim* (**B**) and the fibrosis markers *Col1a1*, *Col1a2*, *Timp1*, *Pdgfb*, and *Pdgfrb* (**C**) in TC, KC and KTC tumors. Sirius red staining of liver cancer tissues (ca) and paracancerous tissues (pc) from TC, KC and KTC mice (**D**). Data are represented by the mean ± SEM. One-way ANOVA was used in **A-D**. *$p < 0.05$; **$p < 0.01$; ***$p < 0.001$.

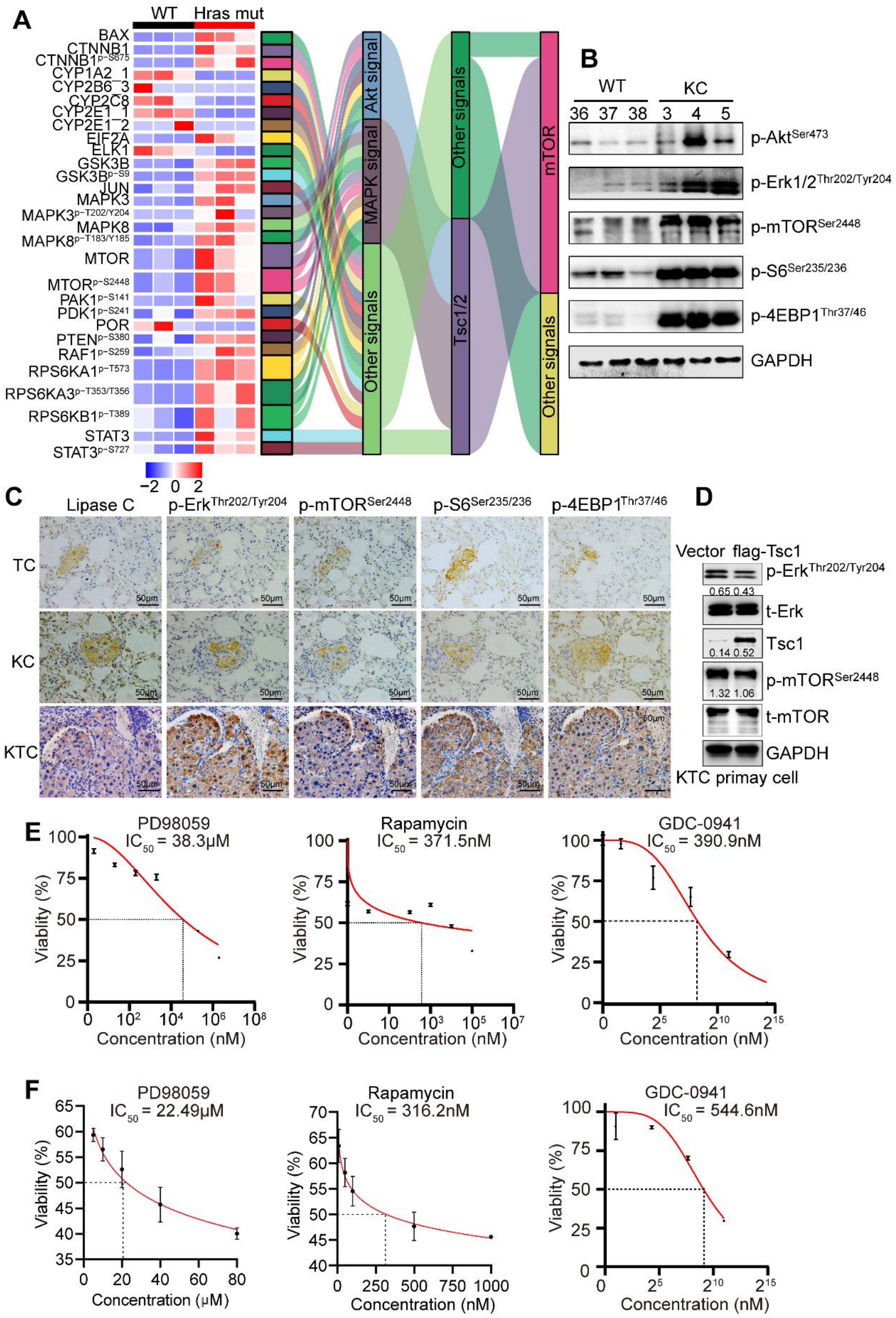

Figure S3. Ras mutation promotes Akt, Erk, and mTOR pathways activation. (**A**) Left, a heatmap showing the differential protein expression of wild-type and Ras mutation liver tumor in proteome sequencing data GSE51357. Right, Sankey diagram illustrating main signals containing differential proteins contributing to mTOR

pathway. (**B**) WB analysis showing the elevated phosphorylation levels of p-Akt^{Ser473}, p-$Erk1/2^{Thr202/Tyr204}$, p-$mTOR^{Ser2448}$, p-$S6^{Ser235/236}$ and p-$4EBP1^{Thr37/46}$ in KC tumors (#3, 4 and 5) compared to normal tissues from WT (wild type) mice (#36, 37 and 38). (**C**) IHC showing the Mek/Erk/mTOR signaling and its downstream p-$S6^{Ser235/236}$ and p-$4EBP1^{Thr37/46}$ were hyperactivated in KTC lung metastatic mets compared with TC and KC lung metastasis mets. (**D**) Reconstitution of Tsc1 in KTC-derived cell lines significantly downregulated the p-$Erk1/2^{Thr202/Tyr204}$. The relative band intensities were added below the bands. (**E-F**) IC50 of PD98059 (Mek-i), rapamycin (mTOR-i) and GDC-0941 (PI3K-i) in #1145 and #1375 KTC primary cell lines. Cells were treated with compounds for 48 h.

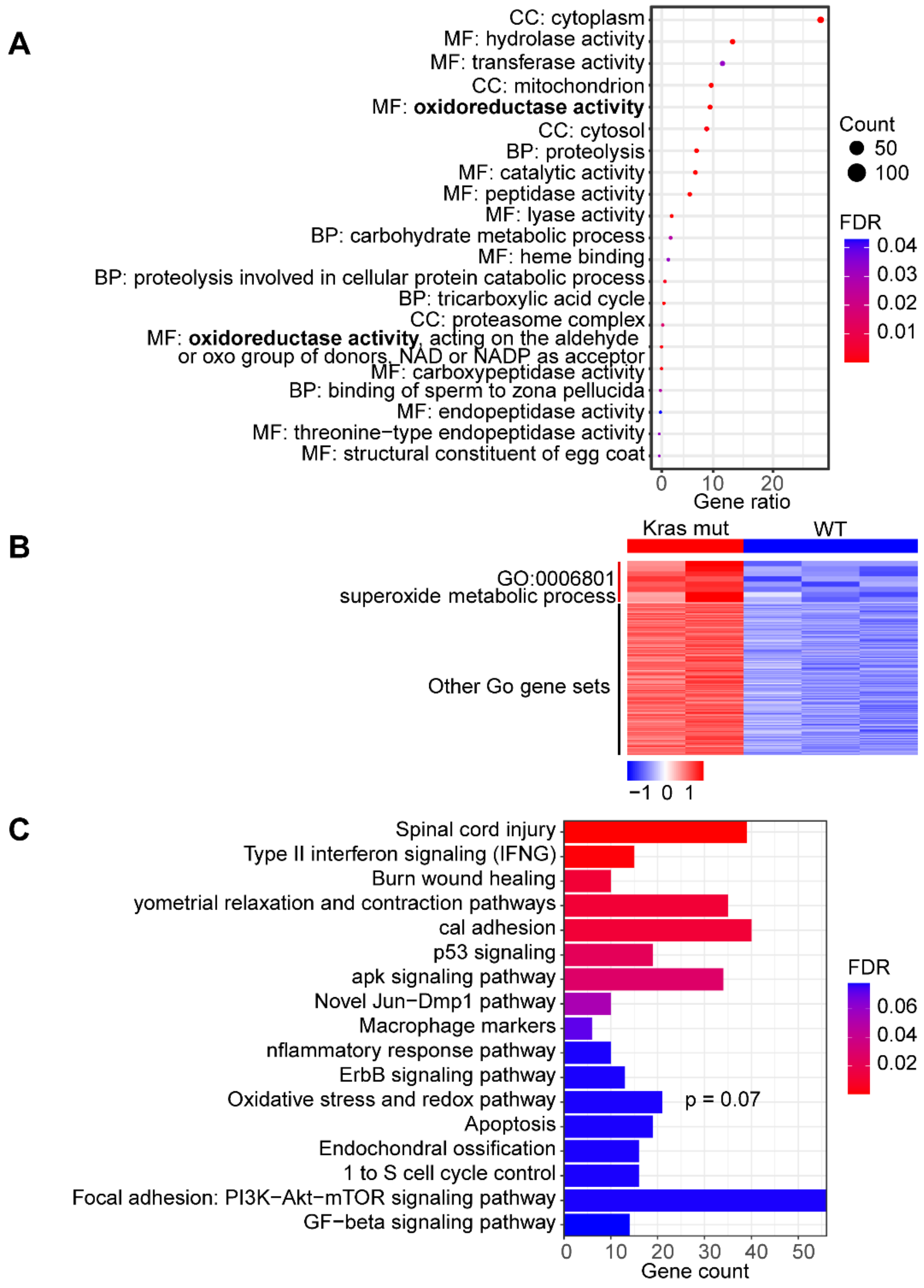

Figure. S4. The potential pathways involved in Kras mutation in liver cancer samples using GSE53630 and GSE105147 cohorts. (**A**) GO analysis using GSE53630 from GEO datasets. Information of molecular function (MF), biological process (BP) and cellular component (CC) was shown in bubble plot. (**B**) A heatmap showing the different expression of biological process gene sets in GSE105147 cohorts. (**C**) Bar plot showing the Wikipathways enrichment analysis of differentially expressed genes from GSE105147 cohorts.

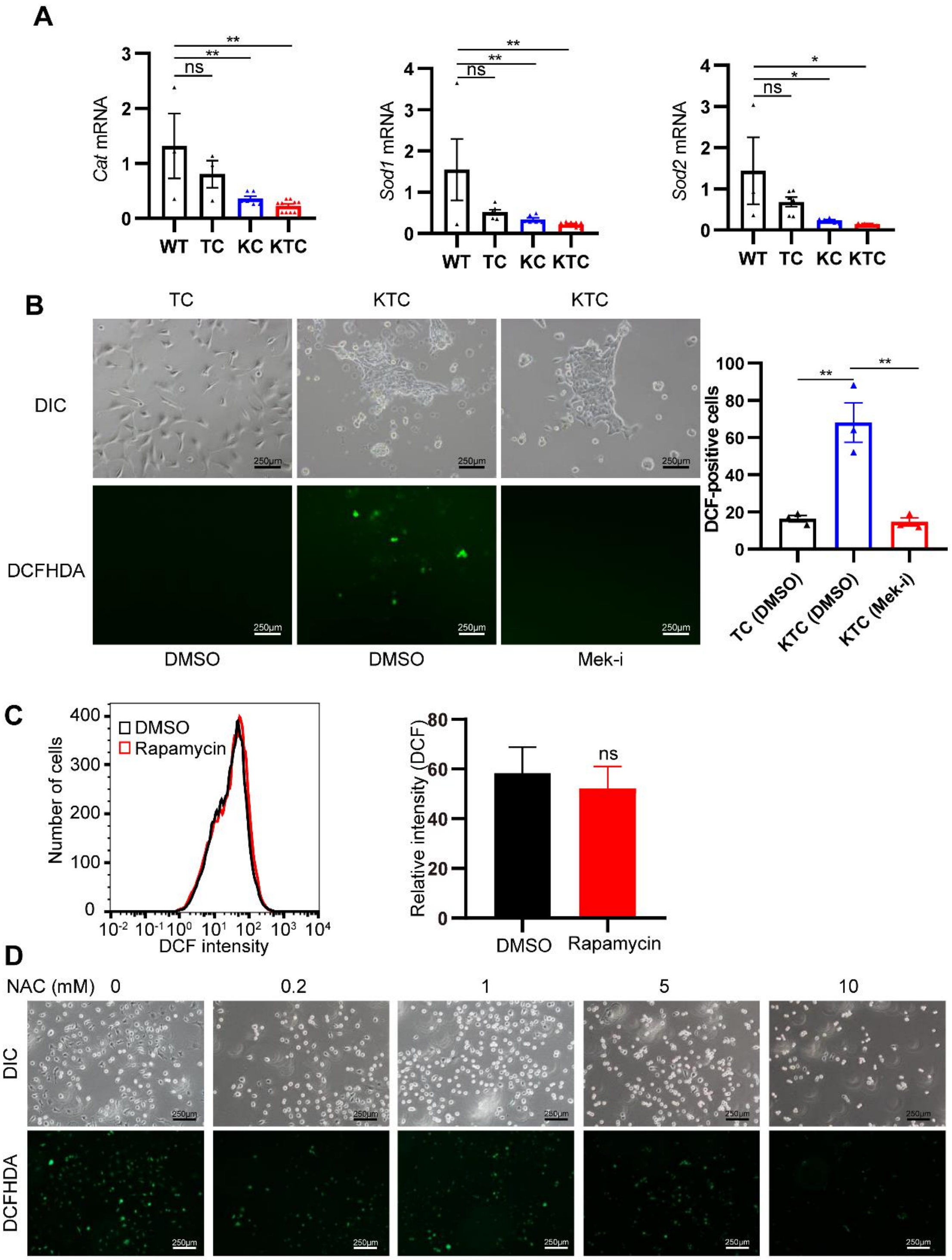

Figure S5. ROS have a high level of accumulation in KTC tumors compared with TC and KC tumors. (**A**) qPCR showing the expression levels of antioxidant markers (*Cat, Sod1* and *Sod2)* in KTC, TC, and KC tumors. (**B**) Representative images showing ROS levels in TC (treated using DMSO), KTC (treated using DMSO) and KTC (treated using the Mek inhibitor PD98059, 20 μM) primary cells. Cells with a positive DCF fluorescence signal were counted among a total of 500 cells on average from 3 independent tumors derived from 3 mice per group. Images were obtained at 10X

magnification; scale bar, 250 μm. (**C**) ROS levels were analyzed by flow cytometry. KTC cells were treated with Rapamycin (100 nM) for 24 h and then stained with DCFHDA (10 μM) for 30 minutes. n = 3. (**D**) ROS levels in KTC primary cells were imaged after exposure to NAC treatment. One-way ANOVA was used in **A** and **B**. Unpaired Student's t-test was used in **C**. ns, no significant. *$p < 0.05$; **$p < 0.01$.

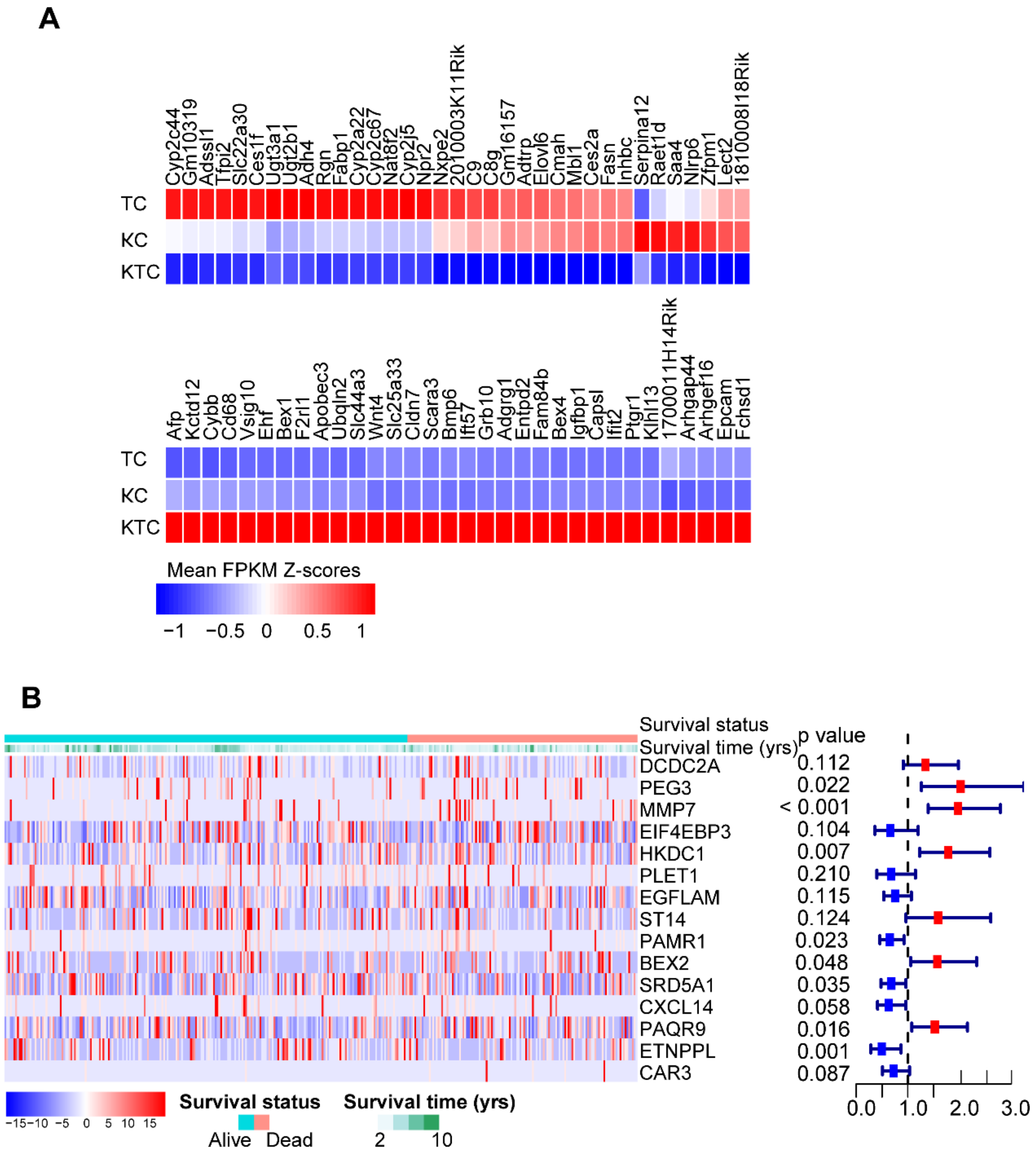

Figure S6. The significant changed genes in KTC, TC and KC mice. (**A**) A heatmap illustrating the top 21-87 significantly changed genes. Each cell representing normalized mean FPKM value of TC KC and KTC groups. (**B**) The relative expression of the 15 genes in TCGA database (top 20 genes without five that cannot be obtained from TCGA database). Left, relative abundance of 15 gene mRNAs. Middle, log-rank test p value of Kaplan-Meier analysis of each gene expression. Right, prognostic risk of each gene mRNA (HCC samples, n = 355). The right red points indicate hazard ratio (HR) for each risk factors and blue points indicate HR for each protect factors; endpoints represent lower or upper 95% confidence intervals (C.I.).

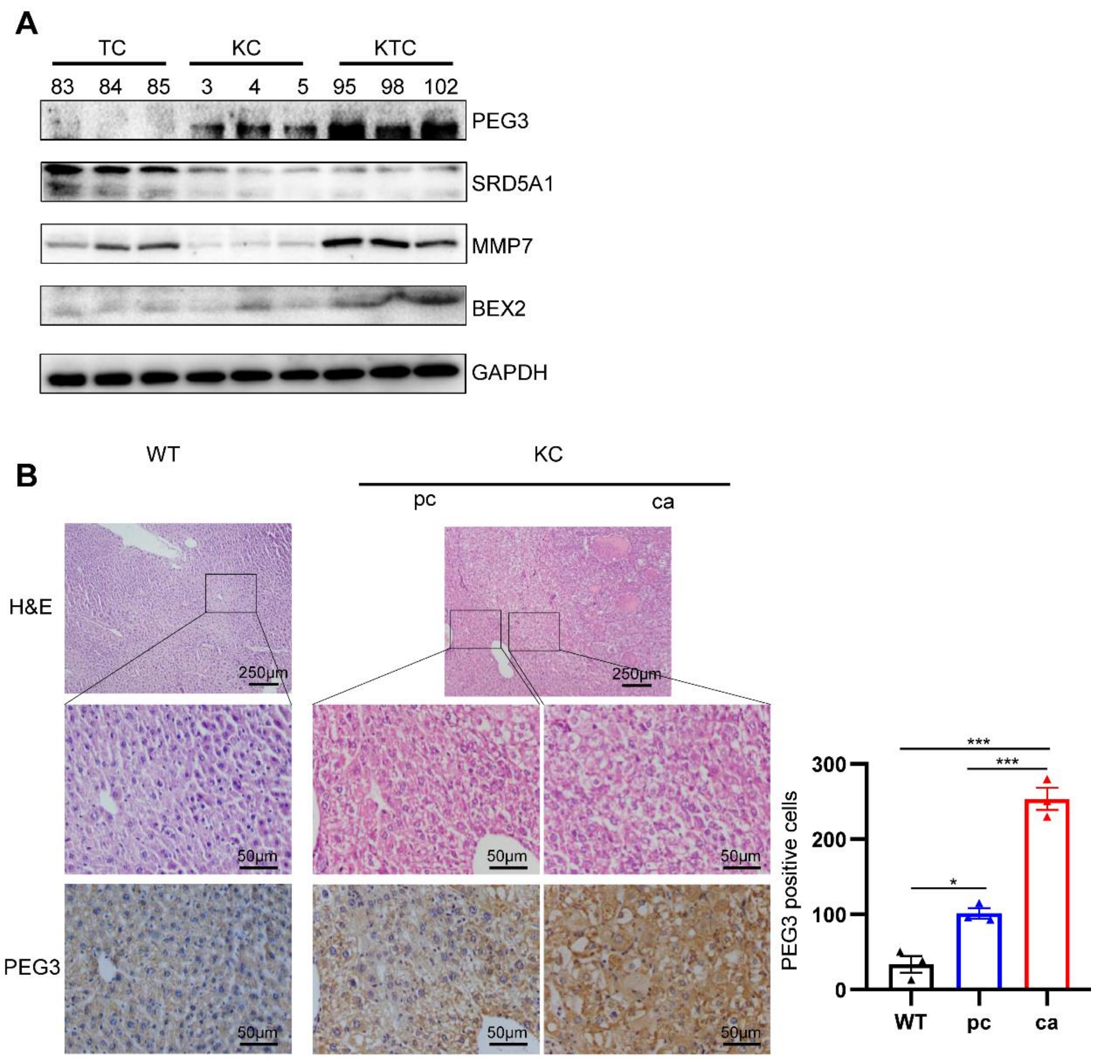

Figure S7. PEG3 is significantly upregulated in tumors from Kras mutant and Tsc1 loss mice. (**A**) The expression of PEG3, SRD5A1, MMP7 and BEX2 at the protein level in tumors from TC (#83, 84 and 85), KC (#3, 4 and 5) and KTC (#95, 98 and 102) mice was analyzed by WB. (**B**) The expression of PEG3 in tumors, paracancerous tissues from KC mice or normal tissues from WT mice was analyzed by IHC. Data are represented by the mean ± SEM. One-way ANOVA was used in **B**. *$p < 0.05$; ***$p < 0.001$.

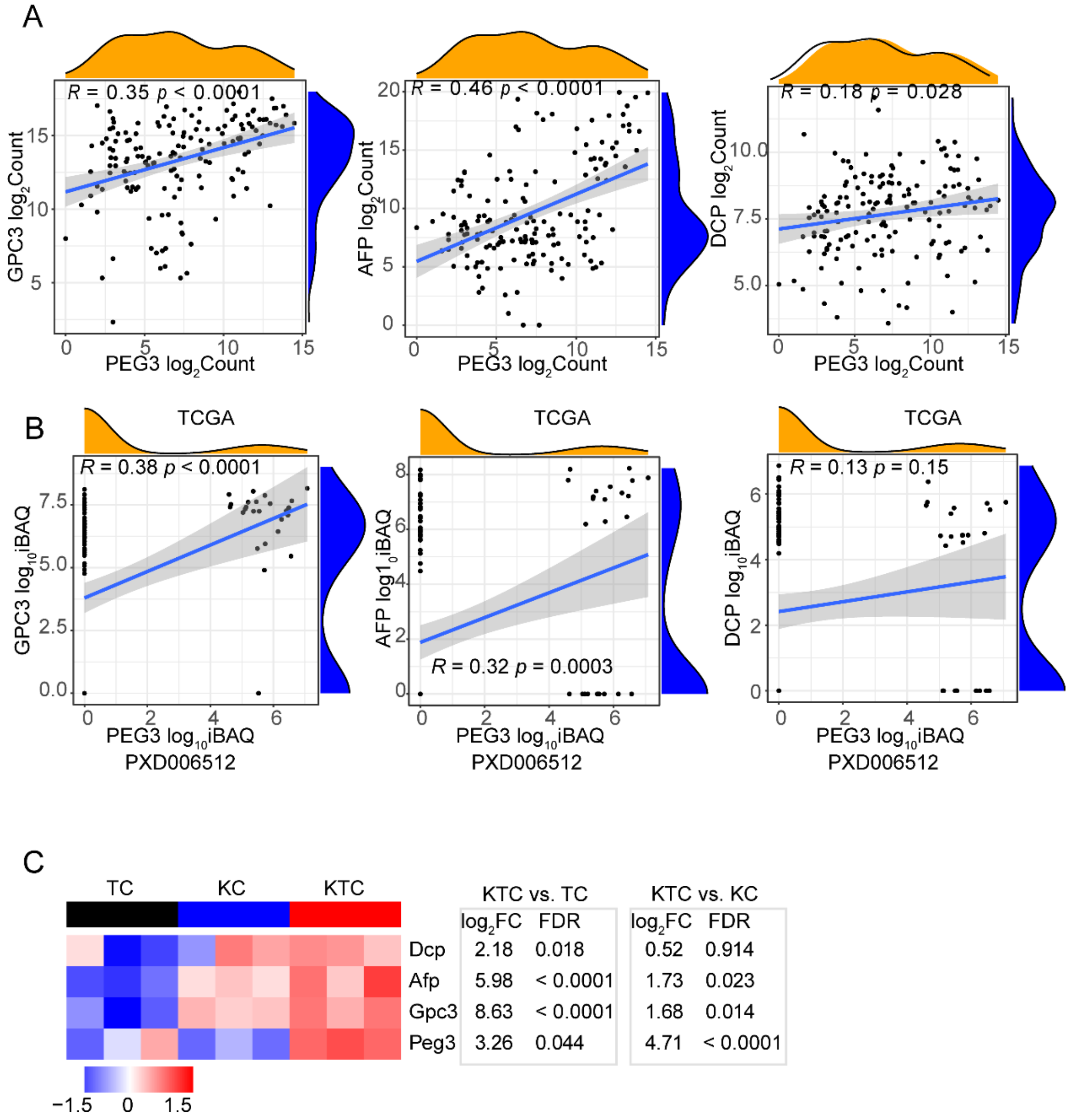

Figure S8. PEG3 expression is positively associated with other HCC biomarkers. (**A-B**) Linear regression model showing the relationship between PEG3 expression and GPC3, AFP and DCP expression based on TCGA database (**A**) and PRIDE database (**B**). (**C**) A heatmap showing the expression of Peg3, Gcp3, Afp and Dcp in TC, KC and KTC tumors. Pearson correlation was used in **A-B**.

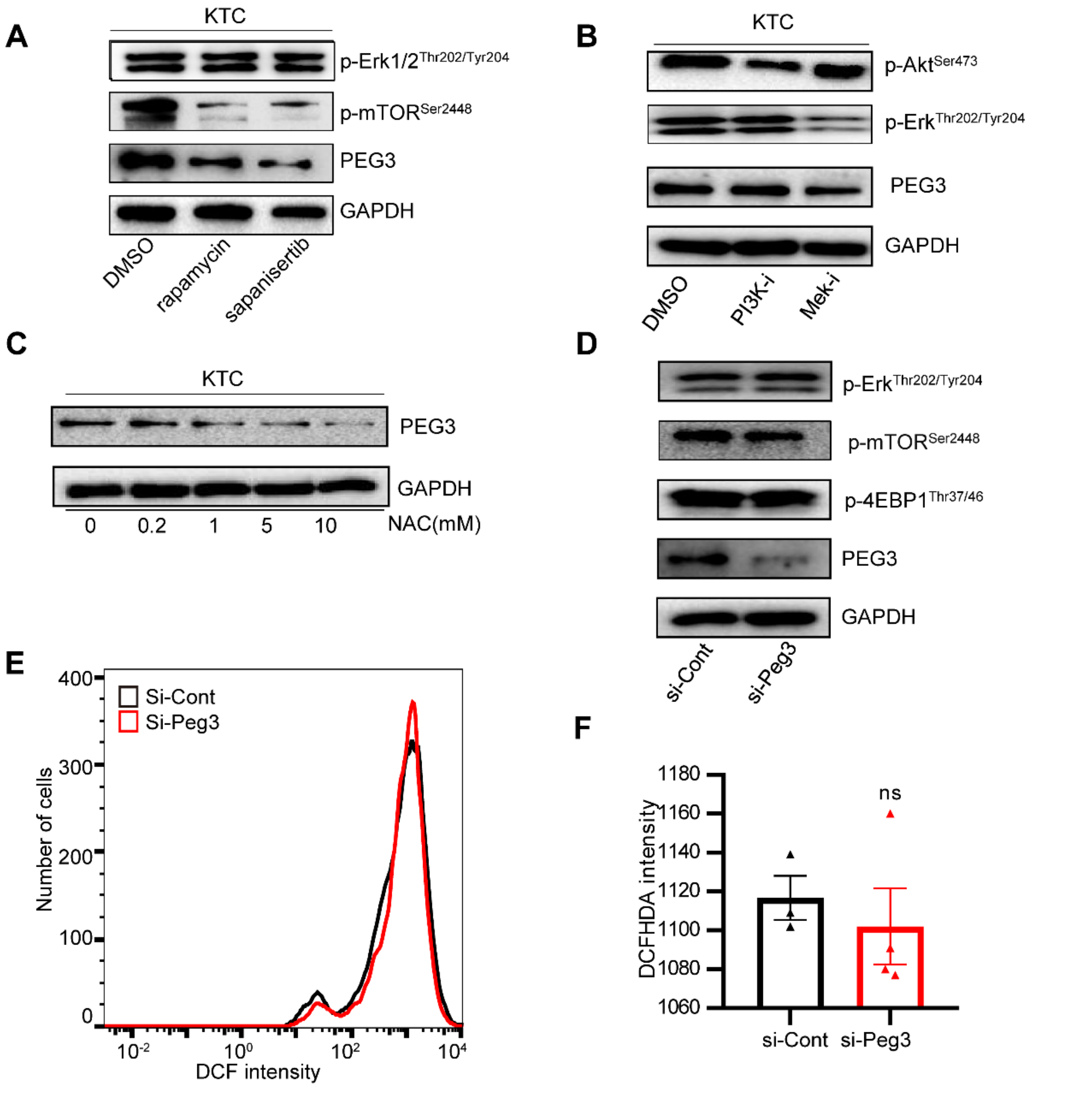

Figure S9. PEG3 is a downstream target of Mek/Erk/ROS/mTOR. (**A**) KTC primary cells were treated with the mTOR inhibitors rapamycin (100 nM) and sapanisertib (1 µM). The expression level of PEG3 was analyzed by WB. (**B**) KTC primary cells were treated with a PI3K-i inhibitor (GDC-0941, 100 nM) and Mek-i (PD98059, 20 µM), and Peg3 was analyzed by WB. (**C**) Primary KTC cells were treated with the ROS antioxidant NAC, and PEG3 was analyzed by WB. (**D**) KTC primary cells were transfected with normal nontargeting siRNA or siRNA targeting Peg3 (si-Peg3), and p-Erk1/2$^{Thr202/Tyr204}$, p-mTOR^{Ser2448} and p-4EBP1$^{Thr37/46}$ were analyzed by WB. (**E, F**) Primary KTC cells were transfected with siRNA control or si-Peg3, and ROS levels were measured by flow cytometry after DCFHDA staining. n = 3. Unpaired Student's t-test was used in **F**. ns, no significant.

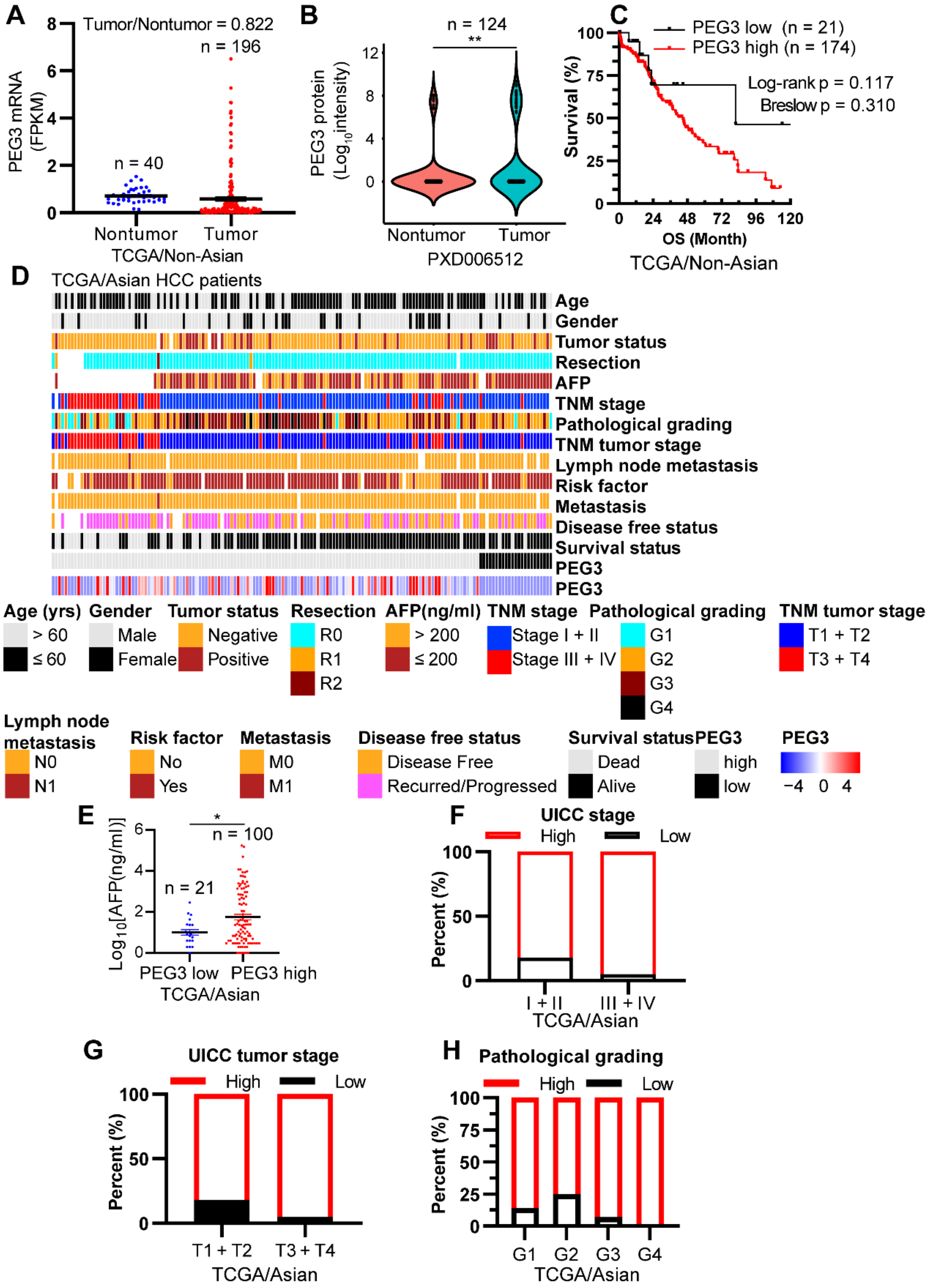

Figure.S10. PEG3 is positively related with poor clinical characteristics of Asian HCC patients. (**A**) Scatter plots showing the expression of PEG3 between TCGA Non-Asian patients' tumor (n = 196) tissues and adjacent nontumor (n = 40) tissues (tumor/nontumor = 0.822). (**B**) Column showing the expression of PEG3 between 124 paired patients' tumor tissues and adjacent nontumor tissues obtained from PRIDE database (PXD006512). (**C**) Kaplan–Meier analysis of the overall survival of TCGA

non-Asian patients with high (n = 174) or low (n = 21) PEG3 expression. (**D**) A heatmap illustrated different expression of PEG3 in each TCGA Asian HCC patients. Each column represents a patient sample and rows indicate clinical characteristics and PEG3 abundance. (**E**) Scatter plots showing the AFP level in TCGA Asian HCC patients with PEG3 high (n = 100) or low (n = 21) expression level. (**F**-**H**) Distribution of PEG3 according to UICC stage (**F**), UICC tumor stage (**G**) or Pathological grading (**H**) in TCGA Asian HCC patients. Data are represented by the mean ± SEM. Unpaired t-test was used in **E**. *$p < 0.05$.

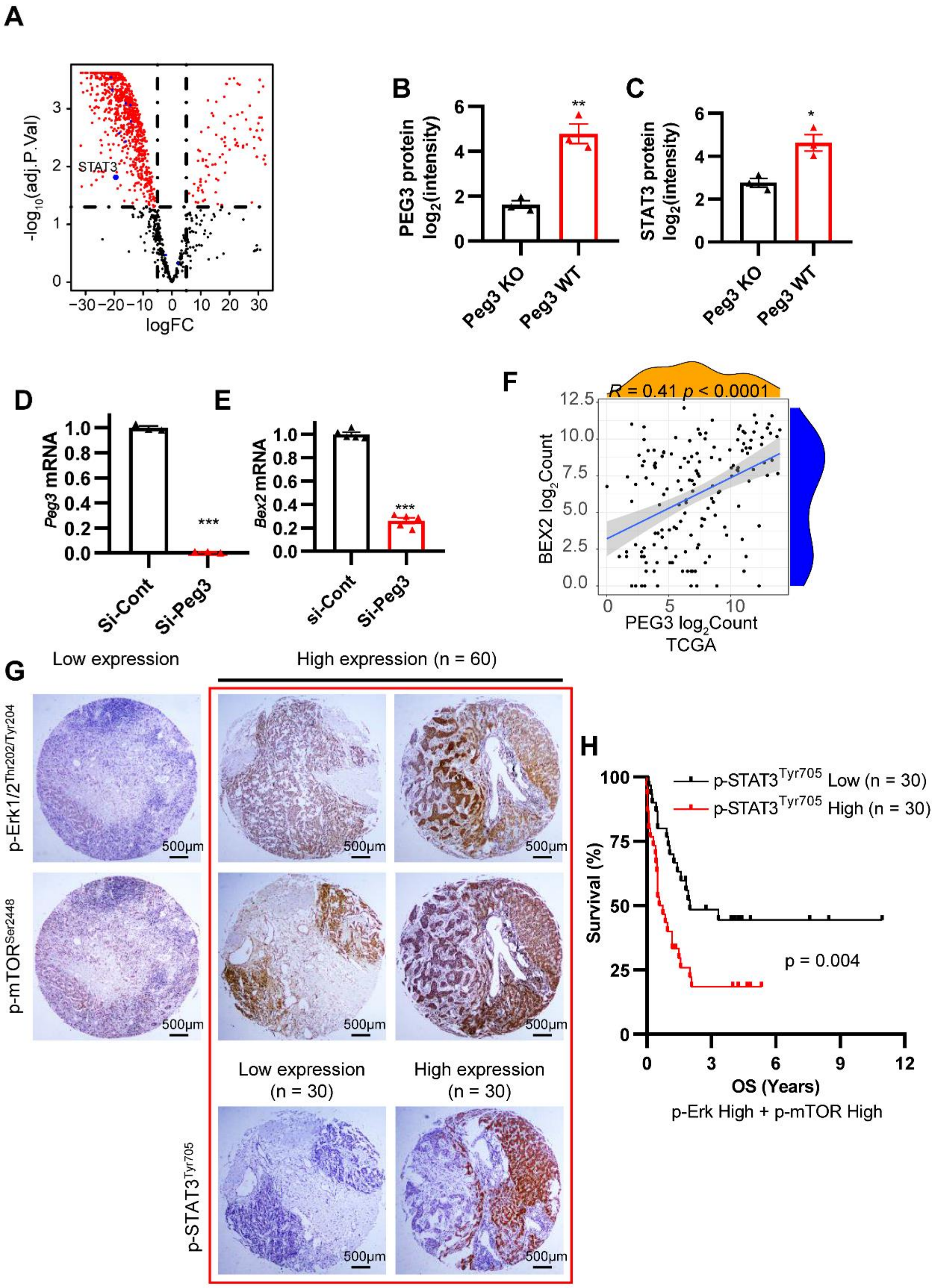

Figure S11. PEG3 positively regulates STAT3 downstream targets BEX2. (**A**) Volcano plot showing differentially expressed proteins identified by co-immunoprecipitation (Co-IP) experiments via tandem mass spectrometry comparing PEG3 knockout (KO) to WT mouse embryonic fibroblast cells. (**B**-**C**) Data showing the protein expression of PEG3 and STAT3 after PEG3 knockdown. (**D-E**) Knockdown of Peg3 on the expression of Peg3 and Bex2 at mRNA levels.

(**F**) Linear regression showing the expression relationship between PEG3 and BEX2 in Asian HCC patients from TCGA database. (**G**) Representative images showing the expression levels of p-Erk1/2$^{Thr202/Tyr204}$, p-mTOR^{Ser2448} and p-STAT3^{Tyr705} in HCC samples. Images were obtained at 4X magnification; scale bar, 500 µm. (**H**) Kaplan–Meier analysis of the overall survival of patients with high (n = 30) or low (n = 30) p-STAT3^{Tyr705} expression in HCC patients with p-Erk1/2$^{Thr202/Tyr204}$ and p-mTOR^{Ser2448} high expression. An unpaired Student's t-test was used in **B**-**E**. Pearson correlation was used in **F**.

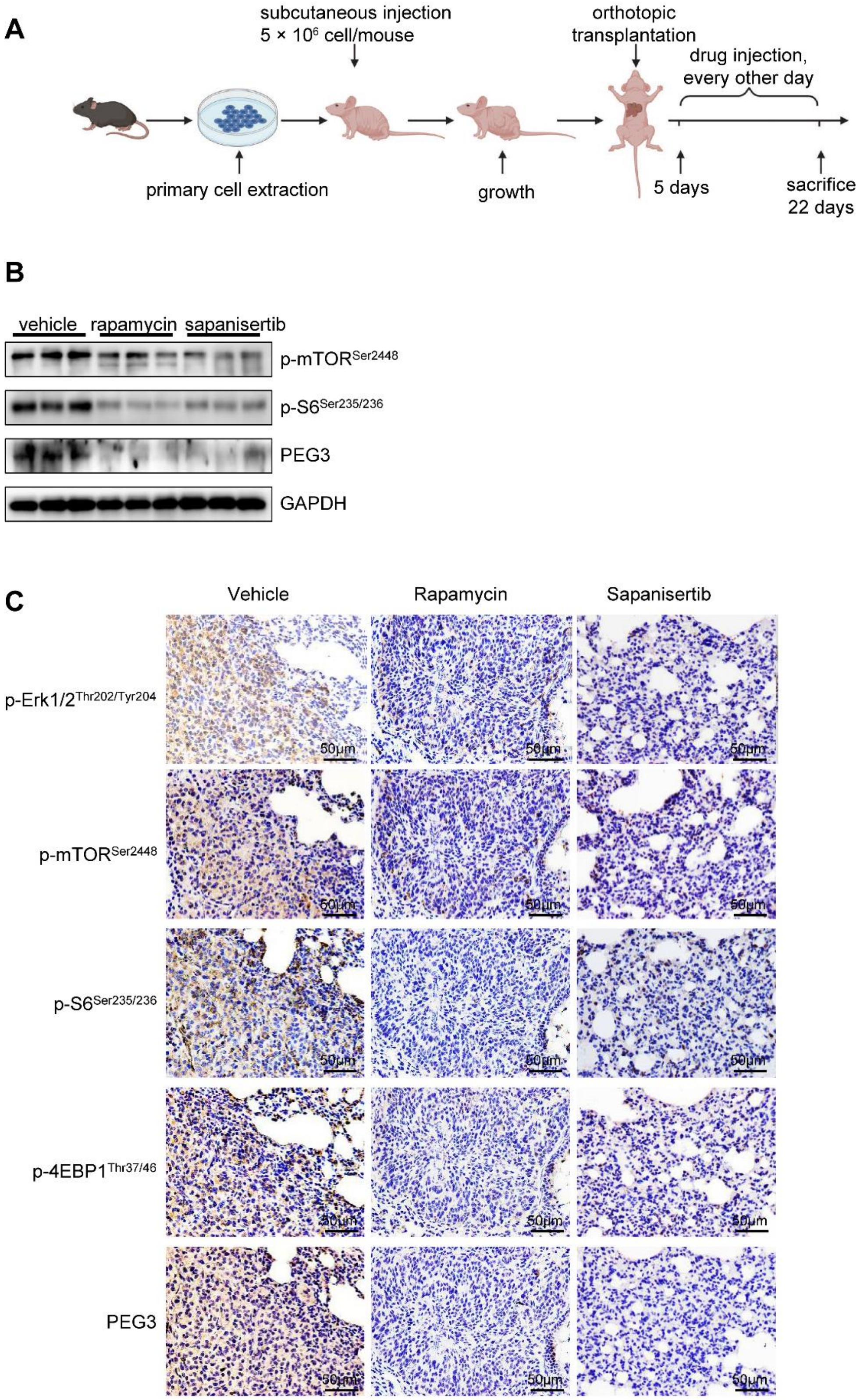

Figure S12. The effects of rapamycin and sapanisertib on Ras/Mek/Erk/mTOR signaling in liver tumors. (**A**) Schematic diagram showing the establishment of the orthotopic HCC model in nude mice and the pharmacological treatment plan. (**B**) WB showing the expression of p-mTOR^{Ser2448}, p-S6$^{Ser235/236}$ and PEG3 in the vehicle group,

rapamycin group and sapanisertib group. (**C**) IHC shown the Mek/Erk/mTOR signaling and its downstream p-S6$^{Ser235/236}$, p-4EBP1$^{Thr37/46}$, PEG3 were decreased in lung metastasis mets of rapamycin group and sapanisertib group compared with vehicle group.

Table S1. Pearson Chi-square test of tumor incidence rate between KTC mice and TC mice or KC mice.

Table S2. Logistic regression of p-Erk1/2$^{Thr202/Tyr204}$ activation and PEG3 expression

Table S3. Logistic regression of p-mTOR^{Ser2448} activation and PEG3 expression

Table S4. Relationship between PEG3 mRNA expression and clinicopathologic characteristics in 157 TCGA/Asian HCC patients

Table S5. Relationship between PEG3 mRNA expression and clinicopathologic characteristics in 195 TCGA/Non-Asian HCC patients

Table S6. Univariate and multivariate analyses indicating the associations between overall survival and various risk factors

Table S7. Primers for qPCR

Table S1. Pearson Chi-square test of tumor incidence rate between KTC mice and TC mice or KC mice.

	with tumor	without tumor	p value
TC	10	6	0.021
KC	8	4	0.035
KTC	11	0	

Statistical analyses were carried out using the Pearson Chi-square test.
*p < 0.05 was considered statistically significant.

Table S2. Logistic regression of p-Erk1/2$^{Thr202/Tyr204}$ activation and PEG3 expression

	**regression coefficient**	**p value**	**Odd Ratio**	**95% C.I.**
**p-Erk1/2$^{Thr202/Tyr204}$**	1.035	**0.001**	2.814	1.500-5.278
**Consistent**	-1.552	**0.002**	0.212	

Table S3. Logistic regression of p-mTOR^{Ser2448} activation and PEG3 expression

	**regression coefficient**	**p value**	**Odd Ratio**	**95% C.I.**
**p-mTOR^{Ser2448}**	0.831	**0.009**	2.296	1.233-4.275
**Consistent**	-1.247	**0.013**	0.287	

Table S4. Relationship between PEG3 mRNA expression and clinicopathologic characteristics in 157 TCGA/Asian HCC patients

**Characteristics**	**N.O. patients (%)**	**PEG3 Low (n = 23)**	**PEG3 High (n = 134)**	**p value**
Gender				
Male	123(78.34)	19	104	0.786
Female	34(21.66)	4	30	
Age(yrs)				
> 60	52(33.12)	11	41	0.149
≤ 60	105(66.88)	12	93	
Metastasis				
M0	150(99.34)	21	129	0.330
M1	1(0.66)	0	1	
Lymph node metastasis				
N0	146(99.32)	20	126	0.289
N1	1(0.68)	0	1	
UICC stage				
I + II	113(72.9)	20	93	**0.041**
III + IV	42(27.1)	2	40	
UICC tumor stage				
T1 + T2	114(72.61)	21	93	**0.040**
T3 + T4	43(27.39)	2	41	
Pathological grading				
G1	14(8.92)	2	12	**0.016**
G2	64(40.76)	16	48	
G3	68(43.31)	5	63	
G4	11(7.01)	0	11	
Risk factor				
Yes	120(82.76)	19	101	0.767
No	25(17.24)	3	22	
Resection				
R0	145(97.97)	23	122	0.751
R1	2(1.35)	0	2	
R2	1(0.68)	0	1	
AFP level				
> 200ng/ml	37(30.58)	1	36	**0.002**
≤ 200ng/ml	84(69.42)	20	64	
Tumor status				
With tumor	30(19.61)	7	23	0.163
Tumor free	123(80.39)	16	107	

PEG3, paternally expressed 3

Statistical analyses were carried out using the Fisher exact test.

*$p \leq 0.05$ was considered statistically significant.

Table S5. Relationship between PEG3 mRNA expression and clinicopathologic characteristics in 195 TCGA/Non-Asian HCC patients

**Characteristics**	**N.O. patients (%)**	**PEG3 Low (n = 21)**	**PEG3 High (n = 174)**	**p value**
Gender				
Male	115(58.97)	15	100	0.249
Female	80(41.03)	6	74	
Age(yrs)				
> 60	130(66.67)	14	116	1.000
≤ 60	65(33.33)	7	58	
Metastasis				
M0	103(97.17)	9	94	0.544
M1	3(2.83)	0	3	
Lymph Node metastasis				
N0	96(97.96)	10	86	1.000
N1	2(2.04)	0	2	
UICC stage				
I + II	129(74.14)	11	118	0.100
III + IV	45(25.86)	8	37	
UICC tumor stage				
T1 + T2	145(75.13)	12	133	0.107
T3 + T4	48(24.87)	8	40	
Pathological grading				
G1	39(20.31)	6	33	0.568
G2	102(53.13)	9	93	
G3	51(26.56)	5	46	
Risk factor				
Yes	131(69.68)	12	119	0.600
No	57(30.32)	7	50	
Resection				
R0	164(92.66)	18	146	0.872
R1	13(7.34)	2	11	
AFP level				
> 200ng/ml	37(25.87)	4	33	1.000
≤ 200ng/ml	106(74.13)	12	94	
Tumor status				
With tumor	76(43.43)	6	70	0.793
Tumor free	99(56.57)	10	89	

Peg3, paternally expressed 3

Statistical analyses were carried out using the Fisher exact test.

*$p \leq 0.05$ was considered statistically significant.

Table S6. Univariate and multivariate analyses indicating the associations between overall survival and various risk factors

Univariables	n	Hazard Ratio (HR)	95.0% CI	p value
PEG3 expression level (Low versus High)	30 versus 30	2.461	1.267-4.777	**0.008**
Gender (Male versus Female)	57 versus 3	0.920	0.222-3.82	0.908
Age (> 60 yrs versus ≤ 60yrs)	53 versus 7	0.791	0.281-2.227	0.657
Histological grading (Well versus Moderate + Poor)	5 versus 55	6.204	0.848-45.407	0.072
UICC stage (I versus II + III + IV)	10 versus 50	5.965	1.434-24.812	**0.014**
Largest tumor size (≥ 10cm versus < 10cm)	13 versus 47	2.490	1.273-4.872	**0.008**
Lymph node metastasis (Negative versus Positive)	57 versus 3	0.801	0.193-3.326	0.760
Vascular thrombosis (Negative versus Positive)	37 versus 23	3.484	1.825-6.652	**< 0.0001**
Intrahepatic metastasis (Negative versus Positive)	37 versus 23	1.793	0.953-3.373	0.070
Recurrence (Negative versus Positive)	18 versus 42	2.843	1.177-6.868	**0.020**
Multivariables				
PEG3 expression level (Low versus High)	30 versus 30	2.040	1.034-4.023	**0.040**
UICC stage (I versus II + III + IV)	10 versus 50		NA	
Largest tumor size (≥ 10cm versus < 10cm)	13 versus 47		NA	
Vascular thrombosis (Negative versus Positive)	37 versus 23	3.060	1.583-5.913	**0.001**
Recurrence (Negative versus Positive)	18 versus 42		NA	

Table S7. Primers for qPCR

	Forward	Reverse
Ki-67	CCTGTGAGGCTGAGACATGG	CCCTCACTCTTGTCAGGGTC
Pcna	AGATGCCGTCGGGTGAATTT	TGTTCCCATTGCCAAGCTCT
Ccnb1	AGCGAAGAGCTACAGGCAAG	CTCAGGCTCAGCAAGTTCCA
Ccnb2	CCGACGGTGTCCAGTGATTT	TTTCTTCGCCACCTGAG
Col1a1	TTCTCCTGGCAAAGACGGAC	CTCAAGGTCACGGTCACGAA
Col1a2	CCAGCGAAGAACTCATACAGC	GGACACCCCTTCTACGTTGT
Timp1	GGCATCTGGCATCCTCTTGT	GGTCTCGTTGATTTCTGGGGA
Pdgfr-β	TCCCACATTCCTTGCCCTTC	GCACAGGGTCCACGTAGATG
Pdgf-β	GGTGAGCAAGGTTGTAATGG	GGAGGCAATGGACAGACAA
Cat	CGGCACATGAATGGCTATGGATC	AAGCCTTCCTGCCTCTCCAACA
Sod1	GGTGAACCAGTTGTGTTGTCAGG	ATGAGGTCCTGCACTGGTACAG
Sod2	TAACGCGCAGATCATGCAGCTG	AGGCTGAAGAGCGACCTGAGTT
Bex2	TCCAAAGTGGAACAAGGCGT	GCACGTAGTAGTCTCCAGCTTC
Peg3	TCATGCACACTAGGGAGAACC	GGCAGCACTCCTACTGAAGG
mmp7	TCGCAAGGAGAGATCATGGAG	CTGCGTCCTCACCATCAGTC
Srd5a1	GAGTTGGATGAGTTGCGCCTA	GGACCACTGCGAGGAGTAG
Icam1	CTGGGCTTGGAGACTCAGTG	CCACACTCTCCGGAAACGAA
Vcam1	CTGGGAAGCTGGAACGAAGT	GCCAAACACTTGACCGTGAC
Mmp9	CCTGGAACTCACACGACATCTTC	TGGAAACTCACACGCCAGAA
Vim	CTGCTTCAAGACTCGGTGGAC	ATCTCCTCCTCGTACAGGTCG
Gapdh	TGGCACCGTCAAGGCTGAGAA	TGGTGAAGACGCCAGTGGACTC

Reference

1. Luo YD, Fang L, Yu HQ, Zhang J, Lin XT, Liu XY, et al. p53 haploinsufficiency and increased mTOR signalling define a subset of aggressive hepatocellular carcinoma. Journal of Hepatology. 2021; 74: 96-108.
2. Zuo B, Qi H, Lu Z, Chen L, Sun B, Yang R, et al. Alarmin-painted exosomes elicit persistent antitumor immunity in large established tumors in mice. Nature Communications. 2020; 11.
3. Raabe EH, Eberhart CG, Harris L, Price A, Yuan M, Arnold A. Synergistic activity of mTORC1/2 kinase and MEK inhibitors suppresses pediatric low-grade glioma tumorigenicity and vascularity. Neuro-Oncology. 2020; 22: 563-74.
4. Grabiner BC, Nardi V, Birsoy K, Possemato R, Shen K, Sinha S, et al. A Diverse Array of Cancer-Associated MTOR Mutations Are Hyperactivating and Can Predict Rapamycin Sensitivity. Cancer Discovery. 2014; 4: 554-63.
5. Chen C, Liu Y, Liu R, Ikenoue T, Guan K-L, Liu Y, et al. TSC–mTOR maintains quiescence and function of hematopoietic stem cells by repressing mitochondrial biogenesis and reactive oxygen species. Journal of Experimental Medicine. 2008; 205: 2397-408.
6. Jin H, Wang C, Jin G, Ruan H, Gu D, Wei L, et al. Regulator of Calcineurin 1 Gene Isoform 4,

Down-regulated in Hepatocellular Carcinoma, Prevents Proliferation, Migration, and Invasive Activity of Cancer Cells and Metastasis of Orthotopic Tumors by Inhibiting Nuclear Translocation of NFAT1. Gastroenterology. 2017; 153: 799-811.e33.

7. Ritchie ME, Phipson B, Wu D, Hu Y, Law CW, Shi W, et al. limma powers differential expression analyses for RNA-sequencing and microarray studies. Nucleic acids research. 2015; 43: e47.

8. Xu Y, Poggio M, Jin HY, Shi Z, Forester CM, Wang Y, et al. Translation control of the immune checkpoint in cancer and its therapeutic targeting. Nature Medicine. 2019; 25: 301-11.

9. Sherman BT, Hao M, Qiu J, Jiao X, Baseler MW, Lane HC, et al. DAVID: a web server for functional enrichment analysis and functional annotation of gene lists (2021 update). Nucleic acids research. 2022.

10. Huang da W, Sherman BT, Lempicki RA. Systematic and integrative analysis of large gene lists using DAVID bioinformatics resources. Nature protocols. 2009; 4: 44-57.

11. Xu J, Ji L, Ruan Y, Wan Z, Lin Z, Xia S, et al. UBQLN1 mediates sorafenib resistance through regulating mitochondrial biogenesis and ROS homeostasis by targeting PGC1β in hepatocellular carcinoma. Signal Transduction and Targeted Therapy. 2021; 6.

12. Perez-Riverol Y, Bai J, Bandla C, García-Seisdedos D, Hewapathirana S, Kamatchinathan S, et al. The PRIDE database resources in 2022: a hub for mass spectrometry-based proteomics evidences. Nucleic acids research. 2022; 50: D543-d52.

13. He H, Ye A, Kim H, Kim J. PEG3 Interacts with KAP1 through KRAB-A. PloS one. 2016; 11: e0167541.

14. Szklarczyk D, Gable AL, Nastou KC, Lyon D, Kirsch R, Pyysalo S, et al. The STRING database in 2021: customizable protein-protein networks, and functional characterization of user-uploaded gene/measurement sets. Nucleic acids research. 2021; 49: D605-d12.

15. Szklarczyk D, Gable AL, Lyon D, Junge A, Wyder S, Huerta-Cepas J, et al. STRING v11: protein-protein association networks with increased coverage, supporting functional discovery in genome-wide experimental datasets. Nucleic acids research. 2019; 47: D607-d13.

16. Shannon P, Markiel A, Ozier O, Baliga NS, Wang JT, Ramage D, et al. Cytoscape: a software environment for integrated models of biomolecular interaction networks. Genome research. 2003; 13: 2498-504.
